# Effect of Panicle Morphology on Grain Filling and Rice Yield: Genetic Control and Molecular Regulation

**DOI:** 10.3389/fgene.2022.876198

**Published:** 2022-05-10

**Authors:** Ajay Kumar Parida, Sudhanshu Sekhar, Binay Bhushan Panda, Gyanasri Sahu, Birendra Prasad Shaw

**Affiliations:** ^1^ Crop Improvement Group, Institute of Life Sciences, Bhubaneswar, India; ^2^ Crop Improvement Division, ICAR-National Rice Research Institute, Cuttack, India; ^3^ Abiotic Stress and Agro-Biotechnology Lab, Institute of Life Sciences, Bhubaneswar, India

**Keywords:** *Oryza sativa* (L.), panicle morphology, ethylene signaling, inter-grain space, cell cycle

## Abstract

The demand for rice is likely to increase approximately 1.5 times by the year 2050. In contrast, the rice production is stagnant since the past decade as the ongoing rice breeding program is unable to increase the production further, primarily because of the problem in grain filling. Investigations have revealed several reasons for poor filling of the grains in the inferior spikelets of the compact panicle, which are otherwise genetically competent to develop into well-filled grains. Among these, the important reasons are 1) poor activities of the starch biosynthesizing enzymes, 2) high ethylene production leading to inhibition in expressions of the starch biosynthesizing enzymes, 3) insufficient division of the endosperm cells and endoreduplication of their nuclei, 4) low accumulation of cytokinins and indole-3-acetic acid (IAA) that promote grain filling, and 5) altered expressions of the miRNAs unfavorable for grain filling. At the genetic level, several genes/QTLs linked to the yield traits have been identified, but the information so far has not been put into perspective toward increasing the rice production. Keeping in view the genetic competency of the inferior spikelets to develop into well-filled grains and based on the findings from the recent research studies, improving grain filling in these spikelets seems plausible through the following biotechnological interventions: 1) spikelet-specific knockdown of the genes involved in ethylene synthesis and overexpression of *β-CAS* (β-cyanoalanine) for enhanced scavenging of CN^−^ formed as a byproduct of ethylene biosynthesis; 2) designing molecular means for increased accumulation of cytokinins, abscisic acid (ABA), and IAA in the caryopses; 3) manipulation of expression of the transcription factors like *MYC* and *OsbZIP58* to drive the expression of the starch biosynthesizing enzymes; 4) spikelet-specific overexpression of the cyclins like *CycB;1* and *CycH;1* for promoting endosperm cell division; and 5) the targeted increase in accumulation of ABA in the straw during the grain filling stage for increased carbon resource remobilization to the grains. Identification of genes determining panicle compactness could also lead to an increase in rice yield through conversion of a compact-panicle into a lax/open one. These efforts have the ability to increase rice production by as much as 30%, which could be more than the set production target by the year 2050.

## Introduction

Rice is a staple food crop satisfying the hunger of the majority of the world population. It contributes greatly in 56% of the world’s calories provided by the cereals in general besides contributing substantially as animal feed (https://www.fao.org/3/Y4683E/y4683e06.htm#TopOfPage). It is grown worldwide and forms the main food crop for more than 50% of the world’s population (Haque et al., 2015). The importance of the rice crop in human life is also reflected from its long process of evolution through domestication for the traits like grain size, grain number, panicle size, grain quality, spikelet fertility, and so forth (Shomura et al., 2008). These traits not only are linked to increasing the production figure but also relate to the quality of the grain produced. With the advancement of science, particularly in the field of genetics, the world rice production dramatically increased in the last half of the 20th century. The achievement was first through the increase in the harvest index by the introduction of the semi-dwarf gene that brought the “green revolution” and second by the production of hybrids in 1970s that exploits heterosis (Xing and Zhang, 2010). This led to doubling of the rice production in 1960s. However, the production must get doubled further by the year 2050 to feed the world’s ever-increasing population (Voesenek and Bailey-Serres, 2009), which would be approximately 9.6 billion during this period (FAO, 2009; Virk et al., 2004). Despite the requirement to scale up the production figure of the crop, it has been hovering at approximately 500 million metric ton (mmt) for the past decade (Shahbandeh, 2021) (Supplementary Figure S1), making achievement of the projected production target nearly impossible. It has also been emphasized that the world’s rice production must increase at least at the rate of 1% per annum to meet the growing demand for food as a result of the ever-growing human population (Rosegrant et al., 1995; Lafarge and Bueno, 2009). In contrast, the annual increase in the production in 2000s has been less than 1% (Normile, 2008), and it has been on a declining trend since the past few decades. The annual increase in the production was 2.7% in 1980s, which decreased to 1.1% in 1990s (Horie et al., 2005). The stagnation shown in the production figure (Shahbandeh, 2021) is a result of such a continuous decline in the annual increment production values over the decades.

Despite stagnancy in the production, rice contributes significantly in the production of cereals all over the world. Its annual production stands at 2679.2 million metric ton (mmt) and is only below maize and wheat in the production figure (OECD/FAO, 2020). However, its cost of production (US$ 428.7/t) is much higher compared to that of wheat (US$ 225.4/t) and maize (US$ 165.2/t) (OECD/FAO, 2020). The high cost of production, nevertheless, does not decrease its importance as a staple cereal as it contributes to 23% of the consumed calories compared to 17% and 10% contributed by wheat and maize, respectively. Thus, the focus of the world and pressure on the researchers is extremely high on pushing up its rate of increase in production substantially so as to meet the projected production target by the middle of this century. The approaches toward increasing the rice yield have been focused on several lines, including breeding, genetics, and biotechnology. The purpose of this review is to bring into lime light such approaches and to provide a direction in which the rice scientists could focus their efforts in enhancing the rice yield based on our current understanding on the subject.

## Ideotype Breeding Approach for Increasing Rice Production

Since the domestication of the crop and the start of agricultural practices that started almost 10,000 years ago, the production of food grains reached to approximately 1 billion tons in 1960, and it took only 40 years to reach the production to approximately 2 billion tons (Khush, 2001). The huge leap in the production of the food grains in the latter half of the 20th century has been possible because of application of the knowledge gained during the period in the agricultural sciences, particularly that related to plant breeding and genetics, and rice as a crop is no exception to this benefit. Almost doubling of the world rice production, from 257 mmt in 1965 to 468 mmt in 1985 (Khush, 1987), has been primarily a result of a well-planned breeding program at the International Rice Research Institute (IRRI), Philipines, and in China. The success came with the development of the semi-dwarf Guang-chang-ai rice variety in 1959 by the transfer of the *Sd-1* gene from Ai-zi-Zhan (Huang, 2001). However, the major success was achieved by the breeders at IRRI through making crosses in 1962 to introduce dwarfing genes from several Taiwanese varieties, including Dee-geo-woo-gen, Taichung Native 1, and I-geo-tse to the tropical tall land races (Peng et al., 2008). The resultant first semi-dwarf, high-yielding modern rice variety, IR8, was released for the tropical irrigated lowlands in 1966 (Khush et al., 2001). The short maturity duration, nearly 110 days, and photo-period insensitivity (Supplementary Figure S2) were the added advantages of the IR8 variety released. The yield potential of the irrigated rice crop was nearly doubled, from 6 t ha^−1^ to 10 t ha^−1^, in the tropics (Chandler, 1982).

In pursuit of increasing the rice yield further, the breeders at IRRI initiated a breeding program for a new plant type (NPT) taking the ideotype breeding approach. “Crop ideotype” is an idealized plant type having all good-to-have features, including efficient photosynthesis, growth, and grain production (Donald, 1968). It was argued that the results of the breeding program would be more fruitful if the desired characteristics are defined beforehand and then the breeding program is initiated to achieve these (Hamblin, 1993). Simulation models predicted that an increase of 25% yield could be possible by modifying certain traits of the current plant type. The modification could be as enhanced leaf growth during the early vegetative stage, reduced leaf growth during the reproductive stage, greater N partitioning to the upper leaf, increased carbohydrate storage capacity in stems, and most importantly a greater reproductive sink capacity (Dingkuhn et al., 1991). Moreover, the increase in sink capacity should accompany an extended period of grain filling (Dingkuhn et al., 1991). Based on the results of the simulation modeling, trait modifications to the high-yielding indica plant type were formulated, which were mostly morphological to make the breeding program relatively easy. The major trait to be introduced in the proposed NPT was to increase the number of grains per panicle up to 200–250 (Peng et al., 1994). Besides, the NPT should possess characters like low tillering capacity; few unproductive tillers; a 90–100 cm plant height; thick and sturdy stems; thick, dark-green, and erect leaves; a vigorous root system; a 100–130 d growth duration; and an increased harvest index (Peng et al., 1994). The NPT lines developed used the bulu varieties or the tropical japonicas and hence are referred to as NPT-TJ. The plant had the ideal features, particularly of the panicle size and spikelet numbers, but showed poor biomass and poor grain filling besides being susceptible to diseases and insects (Peng et al., 2008). The cause-and-effect relationship between low biomass and poor grain filling is yet to be established. However, it was hypothesized that the poor grain filling could be because of compact arrangement of spikelets on the panicle (Yamagishi et al., 1996). Limited success of the first-generation NPT lines led the breeders at IRRI to modify the program and included elite indica parents for crossing with the first-generation NPT lines, and the second-generation NPT lines derived were referred to as NPT-IJ lines (NPT Indica-Japonica). A few second-generation NPT lines had 45–75% greater number of the spikelets than the check variety IR72 and showed a significantly greater yield (Peng et al., 2004). However, if the yield performance of the second-generation NPT lines is compared with the newly developed indica inbred varieties, the yield advantage of the former becomes insignificant (Peng et al., 2004; Yang et al., 2007). Hence, the NPT program of IRRI has largely been of limited success.

The breeding for high-yielding rice varieties, including hybrids, has been a continuous process in China since 1959 (Yuan et al., 1994). In addition, the country formulated the “super rice” or “super hybrid rice” program in 1996 to increase the yield efficiency of the crop through the exploitation of heterosis of the inter-subspecies crosses (Cheng et al., 2007; Peng et al., 2008). Several “super” rice varieties have been released since then, and among them, Xieyou9308 and Liangyoupeijiu are popular because of their good grain quality in addition to the high yield (Peng et al., 2008). The high yield of Xieyou9308 was not only because of the large panicle size but also because of the high grain-filling percentage, 89.6% in the superior spikelets and 80.0% in the inferior spikelets (Wang et al., 2002; Peng et al., 2008).

Both NPT and the “super” rice endeavors although increased the yield of rice significantly; these have not been able to bring a second green revolution in terms of the rice production, unlike that brought by the introduction of the IR8 varieties. The reason for somewhat significant success, although a little, of both the programs was that these avoided the extremes of plant type traits (Belford and Sedgley, 1991) and thus kept the targeted spikelet numbers per panicle to not more than 250 initially and later on not more than 150 spikelets per panicle. The goal was to reduce the percentage of unfilled grains, although the modern generation indica inbred varieties like Upahar show grain filling percentages greater than 90% despite bearing approximately 250 spikelets per panicle (Panda et al., 2015; Sahu et al., 2021). Thus, the strategy adopted for the programs for improving the yield of rice needs a revisit, including the interventions of the modern molecular biology tools.

## Panicle Morphology and Grain Filling

**FIGURE 1:**
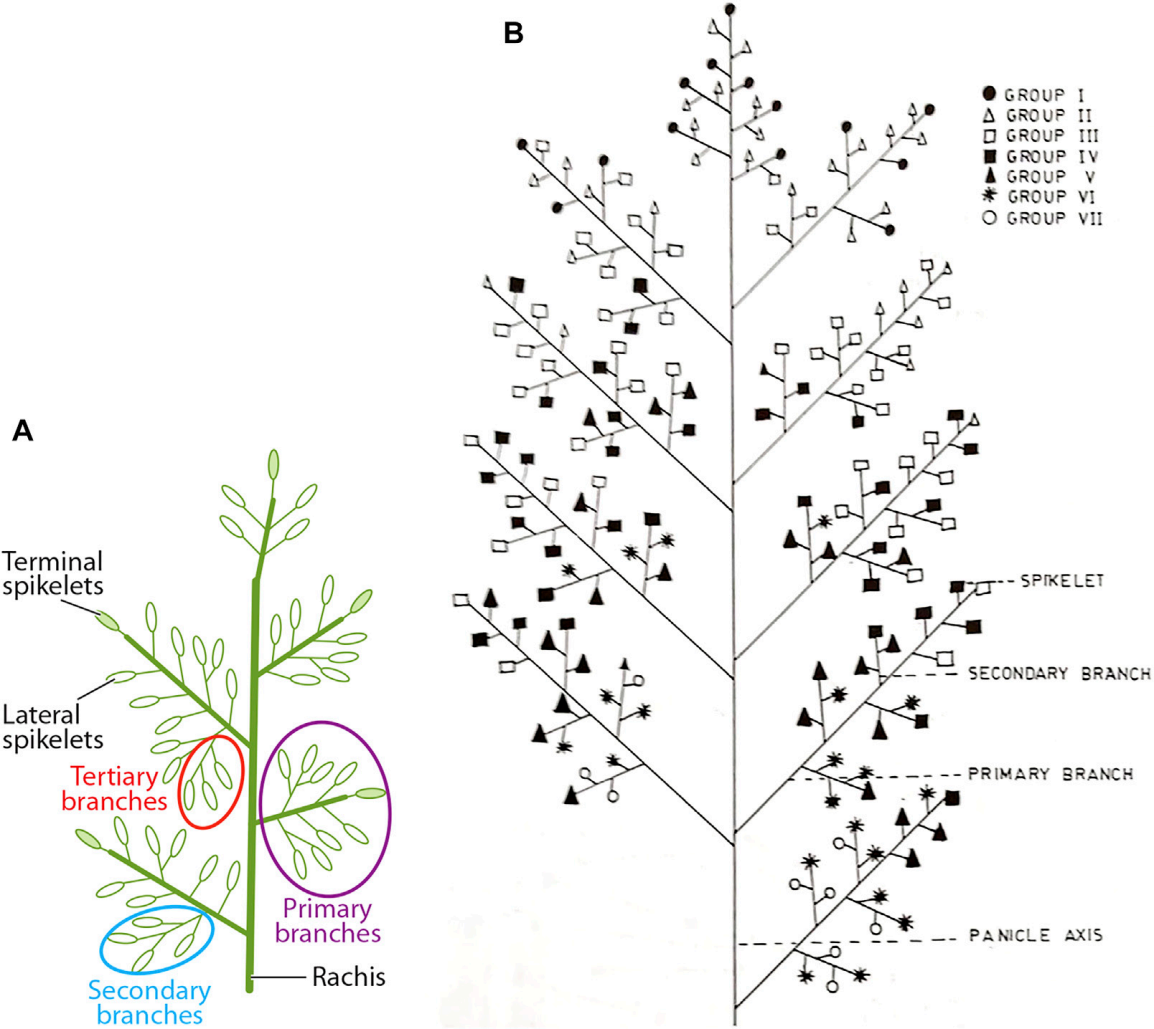
Spatial distribution and development of spikelets on the rachis of the rice panicle**. (A)** The spikelets are termed as terminal or lateral depending upon whether these are derived from the modification of the axis, termed as terminal spikelets, or the lateral branches, termed as lateral spikelets. The lateral branches may be primary, secondary, or tertiary in nature depending upon whether these are originated from the rachis, primary branches, or secondary branches, respectively. **(B)** Pictorial representation of progression of anthesis of the spikelets on the rice panicle, which is an acropetal event with spikelet groups I, II, III, IV, V, VI, and VII reaching anthesis progressively on the first, second, third, fourth, fifth, sixth, and seventh days (Mohapatra et al., 1993). Groups I to III represent the apical (superior) spikelets, while groups V to VII represent the basal (inferior) spikelets. Panel 2A is reproduced with the permission of the author (Xing and Zhang, 2010). Panel 2B is reproduced with the permission of the publisher (Mohapatra et al., 1993).

**FIGURE 2:**
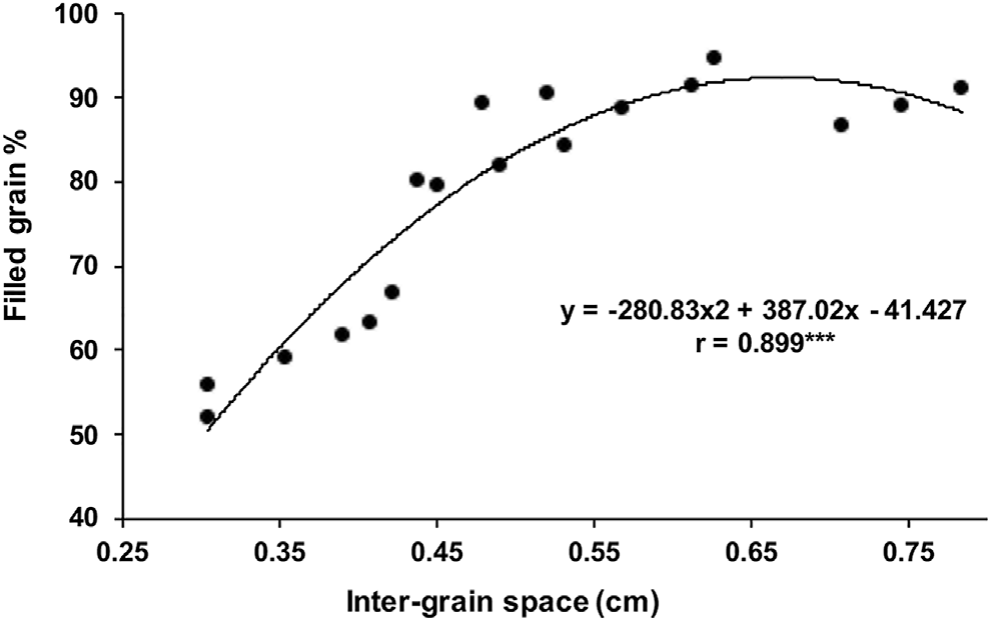
Polynomial regression analysis between the filled grain percentage and inter-grain space. The correlation is significantly positive, and the curve suggests that while the grain filling increases with an increase in the inter-grain space, an inter-grain space greater 0.55 cm is of not much benefit for grain filling.

The main and individual tiller shoots in rice plants are destined to terminate as panicle-type inflorescence having rachis bearing primary, secondary, and even higher-order branches, each getting transformed into a spikelet that harbors a bisexual flower (Figure 1A). The length of the panicle primarily depends on how quickly the rachis gets transformed into a spikelet. The formation of the entire panicle, the juvenile panicle, including the number of spikelets happens inside the boot leaf. The formation of the panicle branches and the spikelets occurs in basipetal succession, that is, the spikelets at the basal region of the panicle are formed earlier than those in the apical region. Thus, the spikelet at the top that is formed by the transformation of the main rachis is the newest. In contrast, the development and maturation of the spikelets start in acropetal succession, that is, from top to bottom. Anthesis of the spikelets progresses slowly from the apical to the basal region and gets completed in approximately 7 days (Figure 2B). Accordingly, the fertilization starts from top to bottom. The well-known phenomenon of apical dominance is maintained in the development and maturation of the spikelets into well-filled grains. The order of dominance recedes from the top to the base. The grain filling also follows the process of apical dominance, with the apical spikelets getting filled first, followed by the filling of the basal spikelets. The apical first principle, however, has great repercussion on filling of the grain in the large-size panicle, such as that developed under the NPT program, where 10–15% of the spikelets, comprising mostly of the basal ones, remain unfilled (Peng et al., 2008). The scenario is even more precarious in the large-size panicles of the indica inbred line bearing 300 or more spikelets where more than 30% of the spikelets remain unfilled (Sekhar et al., 2015a; Panda et al., 2015; Sahu et al., 2021). Thus, the failure of the spikelets to develop into well-filled grains also leads to variation in yield of the rice varieties in addition to the variation created by the number of spikelets per panicle per se. Moreover, the rice varieties bearing larger panicles also show a greater variation in yield within a variety itself compared with the variety bearing small panicles (Yang et al., 2002; Kato, 2004). Although the development of the rice varieties bearing numerous spikelets on the panicle leads to an increase in the sink size, it does not lead to any benefit in terms of the effective yield. The increase in the number of spikelets on a panicle generally leads to a decrease in the inter-grain space, resulting in compactness of the panicle (Sekhar et al., 2015a; Panda et al., 2015; Panda et al., 2016a; Chandra et al., 2021; Sahu et al., 2021). Regression analysis of the relationship between the inter-grain space and grain filling percentage considering several compact- and lax-panicle cultivars has shown that an inter-grain space lesser than 0.55 cm is not favorable for grain filling (Sahu et al., 2021, Figure 2).

Although the basal spikelets in compact panicles remain unfilled, the spikelet thinning treatment, in which some of the apical spikelets are removed, has shown that the basal spikelets are also competent to develop into well-filled grains (Kato, 2004; You et al., 2016). The results of the spikelet thinning treatment thus prove further that the apical spikelets play an inhibitory role in grain filling in the basal spikelets, displaying the phenomenon of apical dominance. Moreover, it is not that the sink becomes a limitation in poor filling of the grains in the compact panicle suggested by Okamura et al. (2018), as the carbon assimilates remain available to the basal spikelets, although these remain unutilized or underutilized (Yang et al., 2006; Yang and Zhang, 2010; Panda et al., 2015). The poor filling of the grains in the basal spikelets despite the availability of the carbohydrate assimilates further ascertains the role of apical dominance in grain development in compact panicles. However, information on the genes controlling the inter-grain space and apical dominance in the metabolism within the inflorescence is scant, knowledge on which would greatly improve the chances of enhancing grain filling in compact panicles. In addition, the plant hormones like auxin, cytokinins, and ethylene also greatly influence the process of grain filling, and hence, spikelet-specific regulation of their contents/presence could be of great benefit in improving grain filling in compact panicles.

## Genetic Perspective of Panicle Morphology

Several genes/QTLs have been reported to influence flower development and/or the panicle architecture in rice (Gupta et al., 2006). However, this review will restrict the discussion only to those related to panicle branching and grain traits that influence the rice yield significantly.

### Grain Numbers and Panicle Branching

**FIGURE 3:**
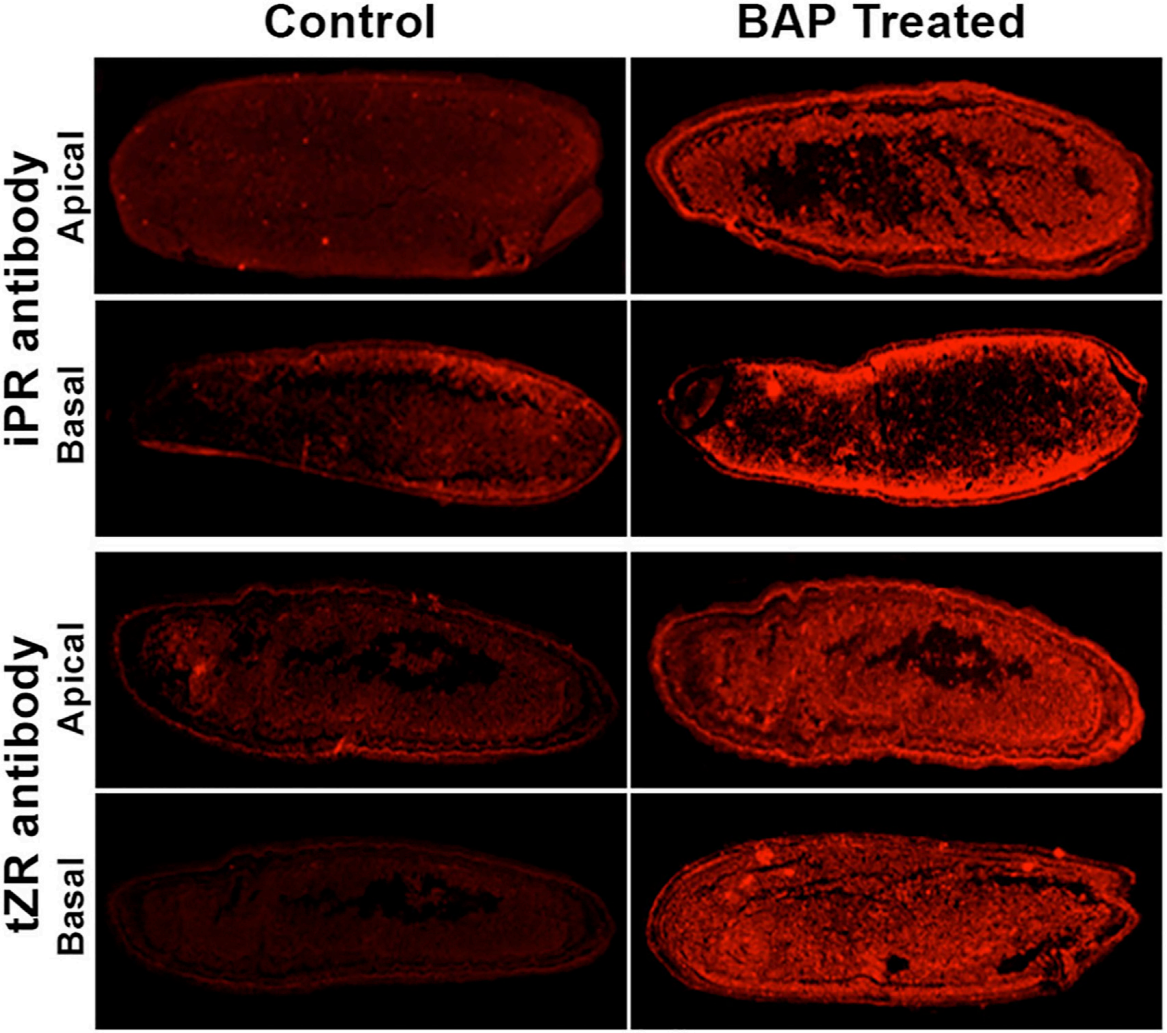
Longitudinal sections of the caryopses sampled from the panicle of the control and that applied with 6-benzylaminopurine (BAP) and stained with the antibody against trans-zeatin riboside (tZR) and N^6^-(Δ^2^-isopentenyl)adenine (iP) riboside (iPR), the precursors of trans-zeatin and isopentenyladenine, respectively, the two cytokinins. The sections were observed under a fluorescence stereo-microscope for the detection of the fluorescence from these antibodies. The caryopses from the basal spikelets of the control plant emitted much lesser fluorescence compared with that sprayed with BAP during the heading. Reproduced with the permission of the author (Panda et al., 2018).

Two genes/QTLs, *Gn1a* (*Grain number 1a*) and *LOG* (*Lonely guy*), both located on chromosome 1, function through regulating the level of cytokinin (Ashikari et al., 2005; Kurakawa et al., 2007). *Gn1a* encodes cytochrome oxidase (OsCKX2) that degrades cytokinin (Ashikari et al., 2005), while *LOG* codes for the protein with phosphoribohydrolase activity that catalyzes conversion of cytokinin nucleotides like iPRMP (*N* ^6^-(Δ^2^-isopentenyl) adenosine-5′-monophosphate) and tZRMP (trans-zeatin-riboside-5′-monophosphate) to their free bases, iP (*N* ^6^-(Δ^2^-isopentenyl)adenine) and tZ (trnas-zeatin), respectively, the metabolically active species of cytokinin (Kurakawa et al., 2007). The loss of function mutation of *Gn1a* leads to an increase in the number of spikelets. The loss of function mutation of *LOG* on the other hand leads to several floral defects, including a decrease in panicle size and spikelet numbers. These mutations indicate the important role of cytokinins in differentiation of the shoot apical meristem (SAM) into inflorescence, including panicle branching and spikelet numbers. This is also reflected from the increase in spikelet numbers upon an increase in iPR (iP-riboside) and tZR (tZ-riboside) levels in the caryopses through aerial application of 6-benzylaminopurine (BAP) (Panda et al., 2018) (Supplementary Table S1, Figure 3). The mechanistic details of the hormone action in the regulation of SAM activity are, however, yet to be known. Besides by the mutation of *Gn1a* and *LOG*, the cellular level of cytokinin is also reportedly regulated by two genes that influence the expression of *OsCKX2*. These are *LP* (*Larger panicle*) and *DST* (*Drought and salt tolerance*), both identified through mutagenesis, with the former as *lp-1* and *lp-2* and the latter as *reg1* (*regulator of Gn1a*). Both *lp* and *reg1* mutants showed a significant increase in the number of both primary and secondary panicle branches and grain numbers (Li M. et al., 2011; Li S. et al., 2013). The *LP* gene was mapped on the short arm of chromosome 2 and identified to code for a kelch repeat-containing F-box protein that interacts with SKP1 (S-phase kinase-associated protein 1) of the SCF (Skp1-Culin-F-box) E3 ligase complex. The *lp* mutants showed a severe decrease in the expression of *OsCKX2*, indicating involvement of LP in modulation of cytokinin equilibrium through direct or indirect regulation of *OsCKX2* expression (Li M. et al., 2011). In contrast to *LP*, *REG1*, mapped on chromosome 3, directly regulates the expression of *OsCKX2* as it encodes a zinc finger protein transcription factor DST that binds to the *cis* element DBS (DST-binding sequence) present in the promoter region of *OsCKX2* (Li S. et al., 2013).

Unlike *Gn1a*, *DEP1* (*Dense erect panicle 1*) is a gain of function mutation that leads to an increase in the number of primary and secondary branches on the panicle, resulting in dense and erect panicles with an increased number of spikelets compared with the wild type (Huang et al., 2009). The mutant (*dep1*) is the dominant allele at the *DEP1* locus on chromosome 9 resulted in by replacement of a 637-bp stretch in exon 5 with a 12-bp sequence, causing a loss of 230 amino acid residues from the C-terminus (Huang et al., 2009). *DEP1* codes for a phosphatidylethanolamine-binding protein (PEBP)-like domain protein, the role of which in panicle development is not known yet, and so also the role of the mutated protein. Similar to *DEP1*, mutation in another gene, *FUWA*, which encodes an NHL domain-containing protein, leads to its premature truncation, resulting in dense and erect panicles, although the number of secondary branches is reduced (Chen et al., 2015). The dense panicle morphology has been stated to be as a result of increased cell division, including in the hull, that is otherwise restricted by *FUWA* (Chen et al., 2015).

Mutation in three more genes/QTLs, *EP2* (*Erect panicle 2*), *EP3* (*Erect panicle 3*), and *qPE9-1*, leads to an erect morphology of the panicle. *EP3* was identified through chemical mutagenesis and mapped to the short arm of chromosome 2 (Piao et al., 2009). The gene encodes an F-box protein, and the mutation, a single base pair change (G/C to A/T), leads to dense and erect panicles, but with a reduced number of spikelets (Piao et al., 2009). In contrast, the gene *EP2*, which encodes a novel protein of unknown functions, results in dense panicles with an increase in the number of spikelets (Zhu et al., 2010). Map-based cloning revealed *EP2* to be located on the long arm of chromosome 7. The mutation of the candidate gene of a major QTL present on chromosome 9, *qPE9-1*, and which codes for a keratin-associated protein, on the other hand, converts a drooping panicle to an erect type without any significant change in the spikelet number (Zhou et al., 2008). The mutation was a result of one single-nucleotide polymorphism (SNP), cytosine-to-tyrosine, and one InDel in the coding region (Zhou et al., 2009). The mechanistic details of action of all these genes are yet to be known, although functionally characterized.

*APO1* (*Aberrant panicle organization 1*) and *SPIKE* (*Spikelet number*) are two genes that influence the spikelet numbers without affecting the panicle compactness much, and unlike *Gn1a*, these are not known to be related to panicle development in any way. The *APO1* locus has been mapped on chromosome 6. The gene encodes an F-box protein, and one nucleotide substitution mutation in it leads to immature transition of the branch and rachis meristem to the spikelet meristem that results in a decrease in the number of spikelets on the panicle (Ikeda et al., 2007). Although its role in inflorescence/panicle development is not known, its overexpression leads to an increase in inflorescence branches and spikelets (Ikeda et al., 2007). Similarly, the overexpression of *SPIKE* in IR64 leads to a significant increase in spikelet numbers, resulting in 13–36% increase in yield over the non-transformed IR64 together with other morphological changes in the plant architecture as pleotropic effects of the gene (Fujita et al., 2013). *SPIKE* was identified as QTL *qTSN4* (total spikelet number per panicle) on the long arm of chromosome 4 of a tropical japonica rice landrace Daringan (Fujita et al., 2013) and was identical to *Nal1* (*Narrow leaf* 1) identified earlier (Qi et al., 2012), suggesting *SPIKE* as an allele of *Nal1* from tropical japonica. *Nal1* is involved in polar auxin transport necessary of differentiation of vascular strands (Fukuda, 2004; Qi et al., 2012), indicating that auxin could influence the spikelet number on the panicle by strengthening the vasculature system. The possible role of SPIKE in vasculature development is also evident from its expression mostly in the vascular bundle at the panicle neck and culm and in young panicles (Fujita et al., 2013).

The time taken for the transition of the vegetative phase to the reproductive phase putatively determines the panicle size and panicle numbers in rice. It is well established that during the development of panicle inflorescence, the shoot apical meristems (SAMs) give rise to primary branches, the SAMs of the primary branches give rise to secondary branches, and so on. At the end, the SAMs of the primary, secondary, or tertiary branches get transformed into spikelets. In *Arabidopsis*, it is known that a delay in the individual transition steps allows a greater time to panicles for development, and this is regulated by terminal flower 1 (*TFL1*) and centtroradialis (*CEN*)-like genes that encode phosphatidyl-ethanolamine-binding proteins (PEBPs) (Bradley et al., 1997; Ohshima et al., 1997). *RCN1* (*Rice centroradialis 1*) and *RCN2* are the putative orthologs of *TFL1*/*CEN* in rice (Nakagawa et al., 2002). Their overexpression in rice produces a significantly greater number of secondary and tertiary branches and three times more spikelets, but with a reduced inter-grain space showing a compact-panicle architecture (Nakagawa et al., 2002).

Two genes, *LAX1* (*Lax panicle 1*) mapped on chromosome 1 (Komatsu K. et al., 2003) and *FZP2* (*Frinzy panicle 2*) mapped on chromosome 7 (Komatsu M. et al., 2003), work together toward the development of panicle branches and spikelets or the inflorescence per se. *LAX1* and *FZP2* code for the bHLH domain and ERF domain-containing protein, respectively, and thus, both are reportedly transcriptional regulators (Komatsu K. et al., 2003; Komatsu et al., 2003 M.). Analysis of their mutants revealed that *lax1* lacked in the development of lateral spikelets, while *fzp2* showed excessive ramification of the rachis branches at the point of initiation of the spikelet meristems without initiation of development of spikelets (Komatsu et al., 2001). The observations led Komatsu et al. (2001) to conclude that while *LAX1* is required for the development of rachis-branch meristems and lateral meristems, *FZP2* specifies them to take the terminal and lateral spikelet identities, respectively.

A recent genome-wide association study (GWAS) has revealed that the number of spikelets on a panicle bears highly significant correlation with the number of secondary branches on it but only to a lesser extent with the number of primary branches (Ta et al., 2018). The variation in the secondary branches explained 89–91% variation in the spikelet numbers, while the variation in the primary branches could explain only 37–42% variation in the spikelet numbers (Ta et al., 2018). The GWAS of Ta et al. (2018) thus supports that the genes like *Gn1a*, *DEP1*, *APO1*, *RCN1*, and *RCN2* could be regulating the number of spikelets by increasing the number of secondary or both secondary and primary branches (Yagi et al., 2001; Nakagawa et al., 2002; Ikeda et al., 2007; Ikeda et al., 2007; Huang et al., 2009), although the mechanistic details of the relationship between the two traits are yet to be understood. However, the genes *Gn1a*, *DEP1*, *APO1*, *RCN1*, and *RCN2* were not detected in the QTLs identified to be regulating the panicle morphology, indicating the involvement of a complex genetic network in the development of the traits determining spikelet numbers; the number of the primary, secondary, and higher-order branches; and the size of the panicle per se (Ta et al., 2018).

A QTL named *WFP* (Wealthy farmers’s panicle) was identified on chromosome 8 by a cross between two japonica rice varieties, Nipponbare and ST-12. The panicle of Nipponbare contained approximately 152 grains and 11 primary branches and that of ST-12 contained approximately 475 grains and 29 primary branches (Miura et al., 2010). The *WFP* QTL carried no gene, but carried *OsSPL14* adjacent to it. The gene expressed much more in the shoot apices and the young panicles in ST-12 compared with Nipponbare (Miura et al., 2010) and hence was assumed to be the real gene regulating panicle branching and spikelet numbers (Miura et al., 2010). The gene *OsSPL14* has also been found to be one among the 12 ORFs identified as the *Ideal plant architecture 1* (*IPA1*) locus present in the QTL *qTn8* mapped on the long arm of chromosome 8. *IPAI* explained 29.9% of variation in the tiller numbers identified through a cross between the indica TN1 or Hui7 and japonica SNJ varieties (Jiao et al., 2010). Cloning and sequencing of *OsSPL14* from TN1 and SNJ revealed that the gene in the latter carried a point mutation that prevented the degradation of its product by the miRNA osa-miR156, which was not there in TN1 (Ta et al., 2018). Thus, *OsSPL14* plays an important role in determining panicle branching and spikelet numbers, the mechanistic details of which are of course yet to be worked out.

The panicle branches and spikelet numbers in rice inflorescence are also reportedly controlled by the *SP1* (*Short panicle 1*) gene mapped on chromosome 11 (Li et al., 2009). The gene encodes a member of peptide transporter family proteins (Li et al., 2009). The inflorescence development in rice occurs in two stages. The first stage involves meristematic activities resulting in branch primordium initiation and spikelet differentiation that gets completed while the panicle is only 4 mm in length or so. The second stage involves elongation of the panicle and panicle branches that ends up with the heading (Li et al., 2009). Mutation in *SP1* leading to 31-bp deletion in the exon causes reduction in the length of the panicle, together with the formation of a much lesser number of primary branches, and drastic reduction in the number of spikelets on the panicle (Li et al., 2009). The mutation causes a drastic decrease in the expression of the gene, the reason for which is yet to be known (Li et al., 2009). The importance of transition of meristematic activity in flower development has been shown in another study where the rice *Supernumerary bract* (*SNB*), identified through a T-DNA insertion mutation study, was found to play an important role in the transition of a spikelet meristem to a floral meristem leading to proper development of the florets (Lee et al., 2007). An AP2 family gene highly homologous to *SNB* was also identified, and since it was closely related to the maize *Indeterminate spikelet 1* (*IDS1*), it was named *OsIDS1* (Lee and An, 2012). A T-DNA insertion mutant line of the gene was also identified, which had a defective floret. Further studies on characterization of the two genes revealed that the *snb osids1* double mutant developed a significantly reduced number of panicle branches and spikelet numbers with simultaneous delay in the transition of the spikelet meristem into the floral meristem, and thus, *SNB* and *OsIDS1* are probably involved in preventing precocious determination of inflorescence and branch meristems (Lee and An, 2012).

### Grain Traits

The yield of a rice cultivar is dependent not only on the numbers of grains produced per unit area but also on the size and weight of the individual grain. The overall size of the rice grain is largely regulated by four genes/QTLs, including *SMG1* (*Small grain 1*), *SMOS1* (*Small organ size 1*), *GS3* (*Grain size 3*), and *GS5* (*Grain size 5*). The *SMG1* locus was found to harbor four open reading frames (ORFs). Out of these, only that encoding mitogen-activated protein kinase kinase 4 (OsMKK4) was found to have a C to T transition in the mutant *smg1* that produced a much smaller size of the grain compared with the wild type, and thus, *OsMKK4* was referred to as the candidate gene of the locus *SMG1* (Duan et al., 2014). The decrease in cell proliferation in the lemma of *smg1* was believed to be the most likely cause of the smaller grain size in it compared with the wild type as the cell length of both the inner and outer epidermal layers was indistinguishable between the mutant and the wild type. The observation was in accordance with the fact that OsMKK4 regulates the expression of the brassinosteroid (BR) pathway-related genes and so also affects BR responses that positively influence cell proliferation, essential for growth of an organ (Duan et al., 2014). In fact, grain size and shape have been reported to be largely determined by cell proliferation in the hull and endosperm (Orozco-Arroyo et al., 2015; Zhang D. et al., 2019). A decrease in endosperm cell division has also been reported to be one among the causes of poor-quality grain formation in indica rice varieties (Sahu et al., 2021). Unlike *SMG1*, the loss of function mutation in *GS3* results in the formation of larger grains (Fan et al., 2006). The QTL *GS3* was identified on chromosome 3 by analysis of the mapping population generated from a cross between the large grain Minghui 63 and the small grain Chuan 7. The *GS3* locus was found to be represented by only one gene encoding a putative transmembrane protein of 232 amino acids. The loss of function was found to be a result of a non-sense mutation in the second exon of *GS3* causing a 178-aa truncation in the C-terminus end (Fan et al., 2006). Mutation in *GS5*, which encodes a putative serine carboxypeptidase, is a result of polymorphism in the promoter region, which leads to a decrease in its expression concomitant with reduction in the grain size (Li Y. et al., 2011). Although the actual function of the gene in regulating the grain size is unknown, a higher expression of *GS5* leads to an enhanced cell division (Li et al., 2009). Further research on *GS5* functions has revealed that GS5 occupies the extracellular leucin reach domain of OsBAK1-7 [brassinosteroid-insentive1 (BRI1)-associated receptor kinase 1–7] and thus competitively inhibits its interaction with OsMSBP1 (membrane steroid-binding protein 1), preventing OsBAK1-7 from endocytosis (Xu et al., 2015). Since BAK1-7 together with BRI1 is involved in perception of BRs, the role of BR signaling in enhancement of cell division by *GS5* may not be ruled out.

Unlike *GS3*, *GL3.1* (*Grain length 3*) regulates the grain length. Two amino acid substitution causes an increase in its phosphatase activity, resulting in the formation of shorter grains in the mutant variety FAZI compared with the wild type WY3 (Qi et al., 2012). *GL3.1* is involved in dephosphorylation of Cyclin-T1;3 and hence influences the cell proliferation; the higher the dephosphorylation, the lesser the cell proliferation and the shorter the grain (Qi et al., 2012). In contrast to *GL3.1*, mutation in *LGS1* (*Large grain size 1*) (Chen et al., 2019) and *GS2 (GRAIN SIZE 2)* (Hu et al., 2015) results in the formation of larger and longer grains. Both encode growth-regulating factor 4 (GRF4), which regulates cell division and the hormonal response pathway, producing pleotropic effects on the panicle morphology, including panicle branching and grain length (Chen et al., 2019). The mutation is in the form of substitution of TC to AA in exon 3 that produces the target site for the miRNA osa-miR396, leading to a decrease in transcript abundance of the genes and so also a decrease in the grain length (Hu et al., 2015; Chen et al., 2019). *GS2* differs from *LGS1* in having other substitutions as well (Hu et al., 2015). Similar to *LGS1*, *SLG7* (*Slender grain on chromosome 7*) and *GW7* (*Grain width 7*) are associated with the formation slender grains (Wang S. et al., 2015; Zhou et al., 2015). The common among the two is that the grain length is positively related with their expression. *SLG7* is homologous to *Arabidopsis LONGIFOLIA1* and *LONGIFOLIA2*, which activate longitudinal organ expansion (Zhou et al., 2015). *GW7* on the other hand encodes a homolog of the *Arabidopsis* thaliana TNNEAU1 (TON1) recruiting motif (TRM) protein, and its expression positively correlates with increased cell division in the longitudinal direction and decreased cell division in the transverse direction in the hull (Wang S. et al., 2015). Thus, both *SLG7* and *GW7* probably provide a slender grain shape by promoting longitudinal cell division in the hull. The promoter of both *SLG7* and *GW7* showed polymorphism in terms of several SNPs and indels between the parents involved in crossing for the generation of the mapping population. Nucleotide sequence analysis of the *GW7* promoter revealed the presence of OsSPL16 binding motifs that were affected because of the indels leading to poor expression of the gene in the long grain variety TaifengA, indicating that OsSPL16 probably controls the grain shape via repression of *GW7* (Wang S. et al., 2015).

Mutation, whether by deletion or by indel, resulting in changes in properties of the protein appears to be a very common cause of changes in the grain shape and size. A great benefit of it is noted in the development of Basmati rice. It is resulted in by a 10-bp indel in the promoter of *GW8* (*Grain weight 8*) that encodes squamosa promoter-binding like protein 16 (OsSPL16). Os*SPL16* is believed to influence the cell cycle machinery and contribute to organ size, and thus, a decrease in its expression causes development of slender grains, much is in demand, compared with the cultivar showing a high expression of the gene (Wang et al., 2012). A point mutation in *TGW6* (*Thousand grain weight 6*) in Nipponbare on the other hand results in a significant increase in grain weight, measured as thousand grain weight (TGW), with respect to that in the Indian landrace Kasalath harboring the active *TGW* gene (Ishimaru et al., 2013). Moreover, it was found that *TGW6* encodes indole-3-acetic acid (IAA)-glucose hydrolase that hydrolyses IAA-glucose to release IAA (Ishimaru et al., 2013), and thus, a greater thousand grain weight in Nipponbare than in Kasalath is linked to the regulation and management of IAA level.

The size of the grain in rice is also regulated by changes in the hull size, as revealed by a few mutation studies. One among them is mutation in *GW2* (*Grain width 2*) that encodes E3 ubiquitin ligase (Song et al., 2007). The gene catalyzes ubiquitination of expansin-like 1 (EXPLA1), a cell wall-loosening protein (Choi et al., 2018). Mutation causes loss of its functions, resulting in an increased cell number in the hull and consequently leading to the formation of a wider hull that allows enhanced filling of the grain milk resulting in the development of heavier grains compared with the wild type (Song et al., 2007). Similarly, mutation in *BG1* (*Big grian 1*), encoding a hypothetical protein, caused by T-DNA insertion in the promoter region of the gene results in a 10-fold increase in its expression that increases the size of the hull as well, leading to an increase in the grain size (Liu et al., 2015). Furthermore, map-based cloning revealed that Nipponbare carries 1212-bp deletion in the region of *qGW5* (QTL for the seed width on chromosome 5) identified in Kasalath that shows a lesser grain width than Nipponbare (Shomura et al., 2008). Through the complementation test, it was found that the 1212-bp deleted region in Nipponbare was associated with its greater grain width than Kasalath (Shomura et al., 2008). It was also found that the greater grain width of Nipponbare was associated with a greater number of the cells in its outer glume compared with that of Kasalath (Shomura et al., 2008), suggesting the size of the hull to be a determinant of size of the rice grain.

The importance of hull size in determining the grain width is also reflected from the identification of two more QTLs, namely, *GW6* (*Grain width 6*) on the short arm of chromosome 6 (Shi et al., 2020) and *GLW7* (*Grain length and weight on chromosome 7*) (Si et al., 2016). The QTL *GW6* harbored five genes, but the promoter of one of them carried four SNPs and one 3-bp (CCT) insertion in the 1-kb region. This mutation was found in the large-grain variety Nan-Yang-Zhan (NYZ) but not in the small-grain variety Hua-Jing-Xian 74 (HJX74), and thus, the concerned gene was called as the candidate gene and referred to as *GW6* (Shi et al., 2020). The cross section of the hull showed a larger outer parenchymal cell size in NYZ than in HJX74 without any difference in the cell number. Several cell expansion-related genes also showed significantly higher expression in the panicle of NYZ compared with HJX74, indicating cell expansion to play an important role in increasing the hull size and so also the grain width (Shi et al., 2020). *GLW7* was identified by GWAS involving 381 japonica varieties of varying grain weights and lengths (Si et al., 2016). *GLW7* harbored 11 genes, but the expression of only one, identified as *OsSPL13*, differed significantly between the small grain and large grain varieties, with the latter showing greater expression than the former, and hence referred to as the candidate gene. The differential expression was a result of a tandem repeat polymorphism in the promoter region of the gene. Furthermore, it was found that the cell density per millimeter in the lemma was significantly higher in *glw7* and the wild-type Dongjing plants (a small seed variety) compared with the Dongjing transgenic lines transformed with the 8-kb genomic sequence of *OsSPL13* from the large grain variety. The opposite was the case for the cell size. These findings strongly suggested that the increase in the grain length and weight associated with *GLW7* was a result of cell expansion rather than any increase in the cell numbers (Si et al., 2016). In addition, the mutation study also ascertains a significant influence of the hull cell length and width on the grain length and width, as was observed in the *wtg1* (*wide and thick grain 1*) rice mutant obtained by gamma ray irradiation (Huang et al., 2017). The mutant produced a wide, thick, and short grain concomitant having shorter and wider cells in the outer epidermis and inner epidermis compared with the wild type (Huang et al., 2017). *WTG1* was found to encode an otubain domain protein (OTUB1) with deubiquitination activity and could be targeting the cell expansion factors, such as SPL13 and GS2 (Huang et al., 2017).

## Grain Filling Biochemistry

**FIGURE 4:**
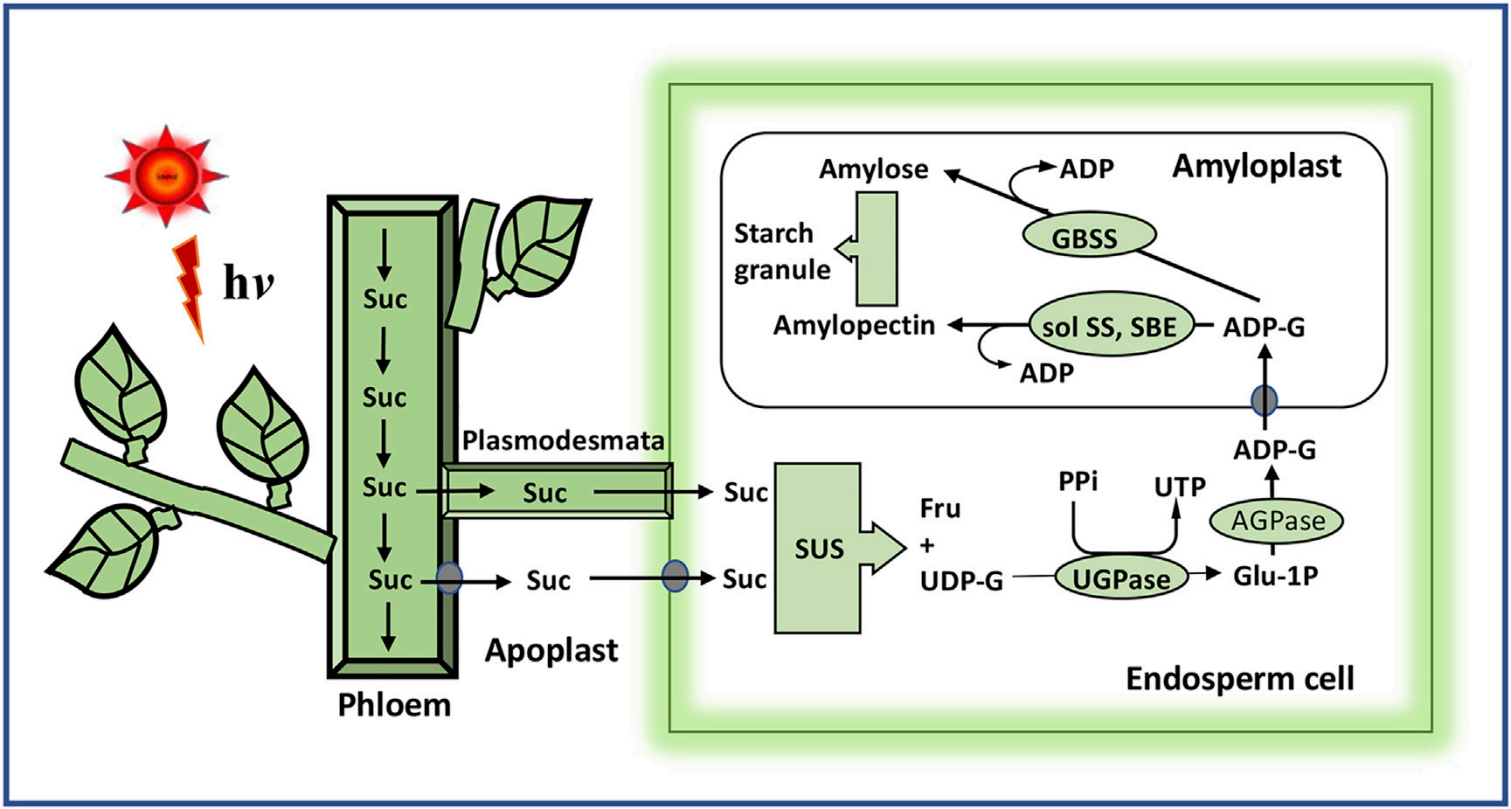
Schematic presentation of starch synthesis from sucrose, the end product of photosynthesis, in the rice grain. Sucrose unloaded from the phloem enters the endosperm cells via the plasmodesmata or through the apoplast with the help of the sucrose transporter. The reactions leading to the synthesis of starch are described in the text. Suc, sucrose; SUS, sucrose synthase; UDP-G, uridine diphosphate glucose; UGPase, UDPG-pyrophosphorylase; AGPase, ADPG-pyrophosphorylase; sol SS, soluble starch synthase; GBSS, granule-bound starch synthase.

The grain filling in rice, or the cereals in general, is a process of systematic deposition of starch in the triploid endosperm cells forming the edible grains. Sucrose from the phloem entering into the endosperm cells is catabolized primarily to uridine diphosphate-glucose (UDP-G) by sucrose synthase (SUS) using its preferred nucleotide UDP (Figure 4). Adenosine diphosphate-glucose (ADP-G) may also be formed utilizing the less preferred substrate ADP. SUS is a very important enzyme of the starch synthesis pathway as the inhibition in expression of its gene by RNAi leads to reduction in starch accumulation to the extent of 40% (Chourey and Nelson, 1976). The overexpression of *SUS* on the other hand increases the accumulation of sucrose significantly (Li J. et al., 2013). UDP-G synthesized is converted first to glucose-1-phosphate (G1P) and then to ADP-G by the action of UDPG-pyrophosphorylase and ADPG-pyrophosphorylase, respectively. Thus, ADPG-pyrophosphorylase, encoded by *GIEF2* (*Grain incomplete filling 2*), plays a crucial role in starch biosynthesis, leading to grain filling, as the further progress in the synthesis of starch depends on the cellular level of ADP-G (Wei et al., 2017). Adenosine triphosphate (ATP) required for driving the second reaction is met from the cytoplasm through the ATP/ADP translocator (Bahaji et al., 2014). ADP-G is joined together by the ∝-1, 4-glucosidic linkage step by step by granule-bound starch synthase (GBSS), leading to the formation of the linear chain of ∝-1,4-polyglucan. Starch-branching enzymes (SBEs) cleave the ∝-1,4-glucosidic linkage and reattach the cleaved fragment with the reducing end to C6 hydroxyl of the glucose moiety of another ∝-1,4-polyglucan chain, creating a branch chain structure, referred to as amylopectin. The elongation of the ∝-1,4-polyglucan in amylopectin is ensured by soluble starch synthase (SS) that adds up the glucose moiety in the ∝-1,4-polyglucan in a fashion similar to GBSS. GBSS elongates only the non-branched ∝-1,4-polyglucans, referred to as amylose. SS and SBEs act in concert to ensure the growth of amylopectin. The starch-debranching enzymes (DBEs), namely, pullalanase and isoamylase, cleave the growing amylopectin to reduce the branching and give a proper shape to the starch crystals being formed. The action of DBEs provides the required hydrophobicity and crystallinity to the starch getting deposited in terms of the edible quality, gelatinization temperature, and cooking time (Fitzgerald et al., 2008).

## Regulation of Grain Filling and Panicle Morphology

Grain filling is a post-fertilization phenomenon. In cereals, primarily it involves development of the triploid endosperm cell formed after the fusion of the central cell with the polar nucleus. The development of the endosperm and embryo goes hand in hand, and the entire ovary develops in caryopsis that matures into a grain. During the course of the development of caryopsis, the endosperm cell divides and redivides and gets filled primarily with starch, referred to as grain filling. The development of spikelets and the panicle as a whole, on the other hand, is a pre-fertilization phenomenon. Both grain filling and panicle development are regulated at the biochemical and molecular levels at several steps, each step being independent of others.

### Endosperm Cell Division and Endoreduplication

**FIGURE 5:**
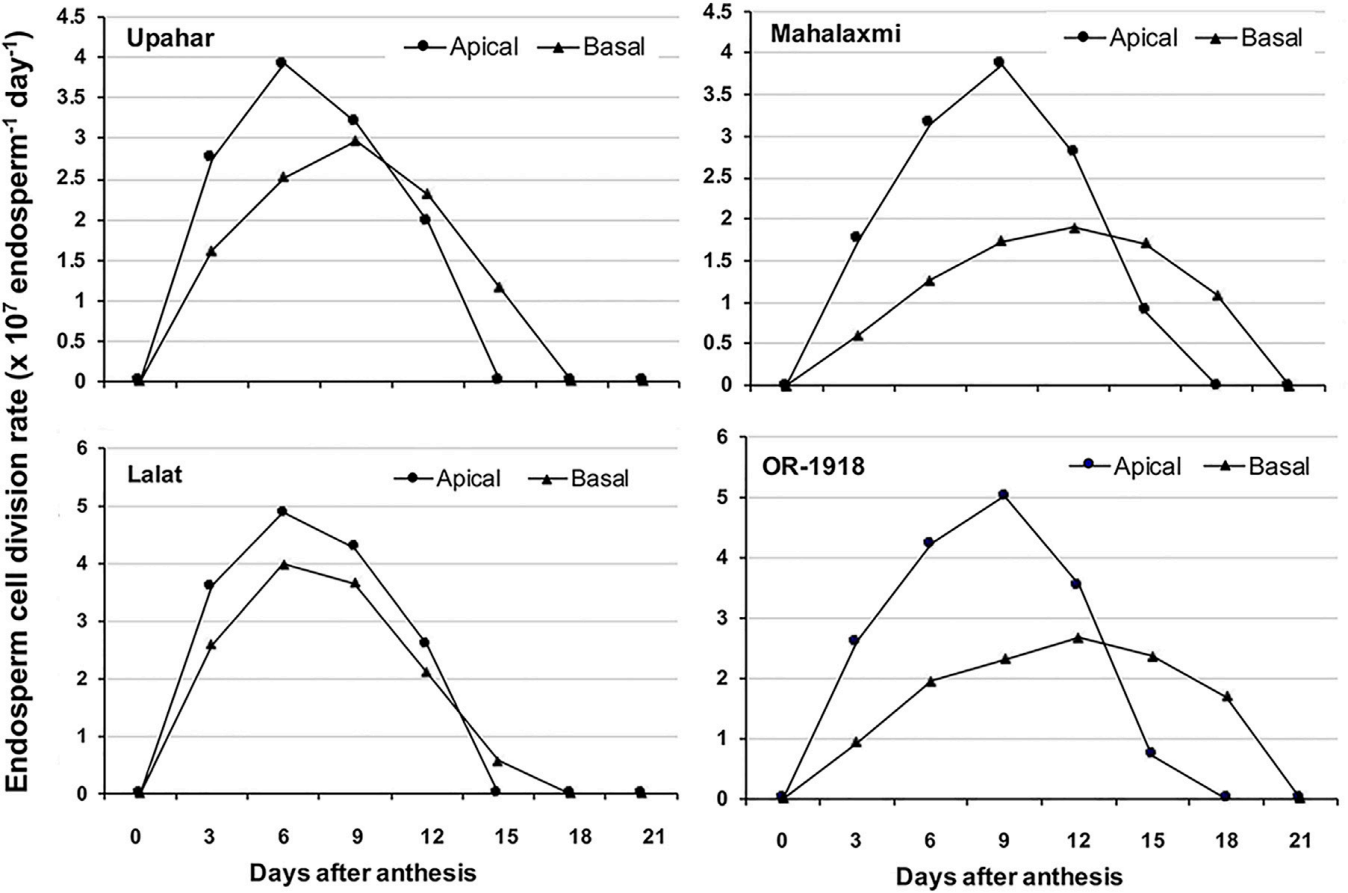
Spatio-temporal depiction of the rate of endosperm cell division in two lax-panicle rice cultivars, Upahar and Lalat, and two compact-panicle rice cultivars, Mahalaxmi and OR-1918. The peak of the endosperm cell division rate in the apical spikelets reaches earlier in the lax-panicle cultivars compared with compact-panicle cultivars, and in the basal spikelets, the arrival of the peak is delayed greatly in the compact-panicle cultivars compared with the lax-panicle cultivars. The cessation of the endosperm cell division in the basal spikelets of the compact-panicle cultivars is also delayed greatly, almost 3 days, when compared with that of the lax-panicle cultivars, leading to poor grain filling in the former. Reproduced with the permission of the author (Sahu et al., 2021).

The first level of control of grain filling occurs at the level of the endosperm cell division. In an ideal situation, the endosperm cell division rate is at peak from 6 to 9 days after fertilization (DAF) and generally ceases after 18 DAF (Panda et al., 2009; Sahu et al., 2021) (Figure 5). It has been reported that the greater and quicker the cell division, the higher is the grain filling in rice (Sahu et al., 2021). The cell division is largely regulated by the cell cycle regulators, and in this context, it has been reported that a slow rate of cell division is linked to a poor expression of *CycB;1* and *CycH;1* (Sahu et al., 2021). Besides, a high expression of *KRP;1* and *KRP;4* also suppresses the cell division in the endosperm (Sahu et al., 2021). The inhibitory effect of KRP on endosperm cell division is also evident from the fact that overexpression of *KRP* decreases cell division (De Veylder et al., 2001; Jasinski et al., 2002; Mizutani et al., 2010).

**FIGURE 6:**
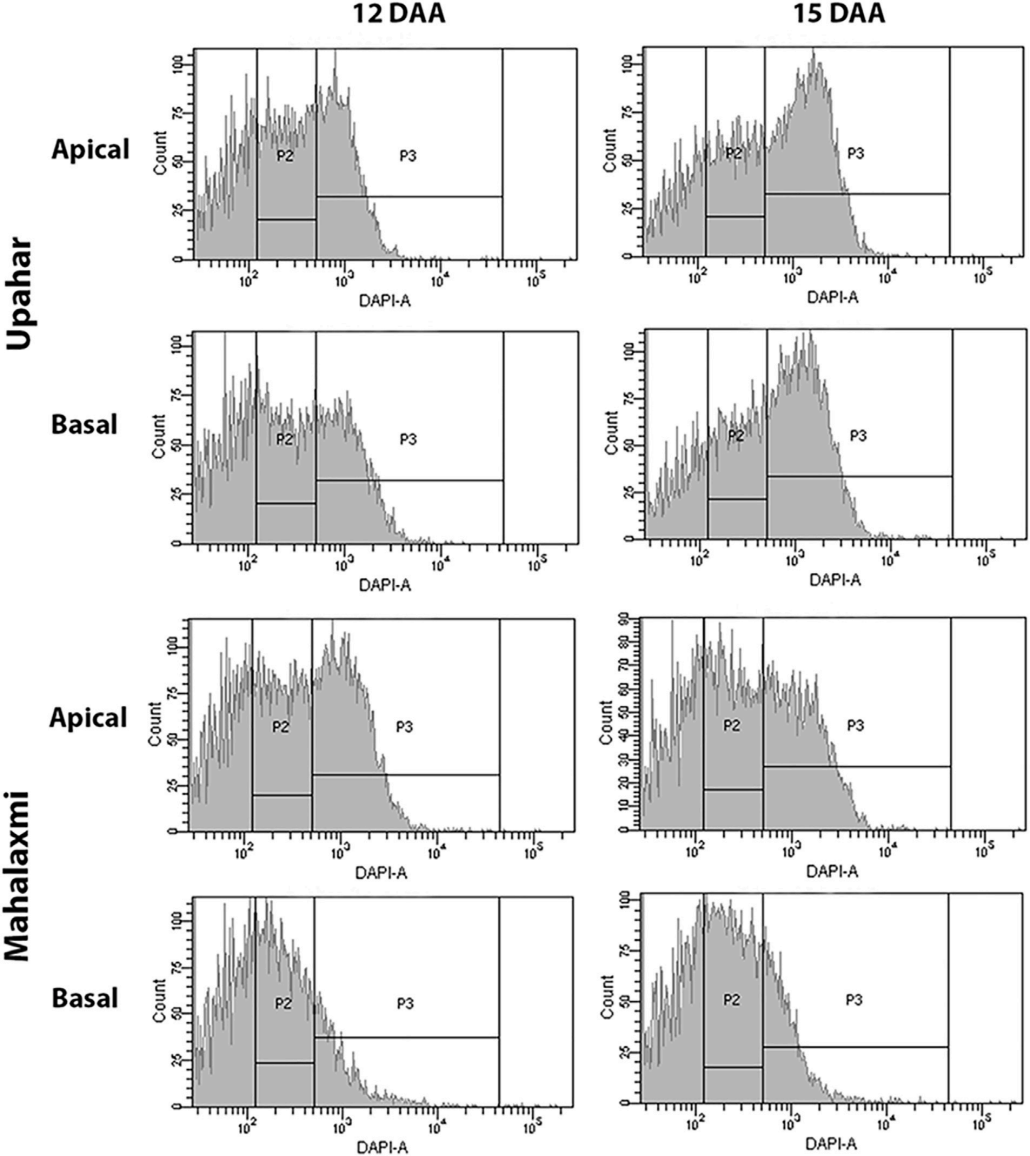
Ploidy status of the endosperm nuclei in the developing caryopses of the apical and basal spikelets during the mid-grain filling stages (12 and 15 days after fertilization, DAF) in a lax-panicle cultivar, Upahar, and a compact-panicle cultivar, Mahalaxmi. The gates P2 and P3 represent the ploidy statuses 3C and >3C, respectively. The ploidy statuses of both apical and basal spikelets in Upahar are more or less similar, whereas they differ greatly in Mahalaxmi with the basal spikelet showing a much lesser number of the endosperm nuclei having the ploidy status of >3C compared with that of the apical spikelets. Reproduced with the permission of the author (Sahu et al., 2021).

Post cellularization of the endosperm and completion of the endosperm cell division, the grain filling is also affected by the level of endoreduplication of the endosperm nuclei. Endoreduplication is a result of sequential and alternate completion of the G- and S-phases of the endosperm cells, but without entering into G2/M transition and karyokinesis, which results in the repeated synthesis of chromatids without their segregation. Endoreduplication thus increases the ploidy status of the endosperm cells, providing a platform for enhanced expression of the genes required for the purpose of grain filling. Studies have shown a positive relationship between endoreduplication and grain filling in rice (Panda et al., 2016a; Sahu et al., 2021) (Figure 6).

### Transcriptional Regulation

The next level of regulation of grain filling occurs through the regulation of expression of the starch-biosynthesizing enzymes by transcription factors. The earliest report in this regard is the regulation of expression of the *Wx* gene (*GBSS1*) by two transcription factors, MYC protein (OsBP-5) and an ethylene-responsive element binding protein (EREBP), OsEBP89. Both bind at the 31-bp sequence ranging from -840 to -810 bases (Yao et al., 1996; Zhu et al., 2003). Within the 31-bp sequence, OsBP-5 binds to the sequence CAACGTG and OsEBP89 binds to the adjacent sequence GCCAAC, and their interaction drives the expression of the gene (Zhu et al., 2003). The expression of the *Wx* gene is also influenced by an NAC transcription factor as *Wx* co-expresses with *NAC26* (Wang et al., 2019). A moderate soil drying condition increases the expression of both the genes significantly with a concomitant increase in the individual grain weight of the inferior spikelets (Wang et al., 2019). Moreover, NAC26 has been shown to interact directly with the promoter of the *Wx* gene (Wang et al., 2019), suggesting direct involvement of the transcription factor in the grain filling process in rice. In addition, NAC has also been indicated to play a key role in activating the expressions of the starch-synthesizing genes in general under moderate soil drying conditions (Wang et al., 2020a), although the details of the mechanism involved are not known. Unlike NAC, the transcription factor OsbZIP58 (basic leucin zipper 58) is known to directly regulate the expression of as many as six starch synthesis pathway genes, including *Wx*, *OsSSIIa, SBEI*, *OsBEIIb, OsAGPL3*, and *ISA2* by binding with the ACGT element in their promoter (Wang et al., 2013). Since the promoter of the *Wx* gene contains as many as 16 ACGT elements, its expression is most affected by OsbZIP58 (Wang et al., 2013).

Furthermore, co-expression analysis has revealed that the protein rice starch regulator1 (RSR1), an APETALA2/EREBP family transcription factor, negatively regulates the expression of many type I starch-synthesizing genes, including that of *OsSS* (starch synthase), *OsBE* (starch-branching enzyme), *OsGBSS* (granule-bound starch synthase), *OsISA* (starch-debranching enzyme: isoamylase), *OsAGPL* (ADP-glucose pyrophosphorylase large subunit), *OsAGPS* (ADP-glucose pyrophosphorylase small subunit), and *OsPHOL* (starch phosphorylase L) (Fu and Xue, 2010). Besides, RSR1 has also been found to regulate the expression of *GBSS1* in rice caryopses (Sekhar et al., 2015a). However, the mechanism underlying downregulation of expression of the starch-synthesizing genes by RSR1 is not yet understood.

Unlike RSR1, two important transcription factors, the rice prolamin-box binding factor (RPBF), which is a DOF (DNA binding with one finger) family transcription factor, and a basic leucine zipper transcription factor, *RISBZ1*, positively regulate the expression of the type I starch-biosynthesizing enzymes (Kawakatsu et al., 2009; Fu and Xue 2010; Schmidt et al., 2014). RPBF and RISBZ1 in fact act synergistically to modulate the expression of the starch-synthesizing genes, as is seen by their overexpression; the two transcription factors overexpressed together in a plant produced greater expression of starch-biosynthesizing enzymes compared with the sum total of the expression produced by their individual overexpression (Yamamoto et al., 2006).

Grain filling and seed development in rice are affected not only by the activities of the starch-biosynthesising enzymes but also by the factors that determine the size and shape of lemma and palea, the hull. It has been seen that the atypical basic helix-loop-helix (bHLH) gene (Os03g0171300) having no DNA binding domain expresses in a high amount in the hull (Heang and Sassa, 2012a). The overexpression of the atypical bHLH leads to an increase in length and weight of the rice grain, and hence, it is named *Positve regulator of grain length 1* (*PGL1*) (Heang and Sassa, 2012a). Subsequently, another atypical bHLH (Os02g0747900) was functionally characterized and named *PGL2* (Heang and Sassa, 2012b). It is known that atypical bHLH proteins act as inhibitors of typical bHLH proteins that function as transcription factors since these have a DNA-binding domain as well along with the helix-loop-helix region (Toledo-Ortiz et al., 2003). The inhibition occurs through heterodimer formation. The interacting partner of PGL1 and PGL2 was identified and named APG (antagonist of PGL1/2) (Heang and Sassa, 2012a; Heang and Sassa, 2012b). As the overexpression of *PGL1*/*PGL2* led to an increase in length and width of the rice grains, *APG* was considered as a negative regulator of rice grain length and width. This was proved by generating knockdown of *APG* by RNAi that showed the formation of longer grains than the wild type (Heang and Sassa, 2012a; Heang and Sassa, 2012b). Furthermore, it was observed that the formation of the longer grain in the transgenic plants was associated with an increase in length of the inner epidermal layer cells, indicating the size of the hull as an important determinant of the size of grains. However, the information on the expression of the genes influenced by APG is scant.

Three plant-specific transcription factors, namely, OsSPL13, OsSPL14, and OsSPL16, have also been observed to play diverse roles in determining the panicle morphology, without influencing the grain filling biochemistry. OsSPL13 is encoded by *GW7*, and it promotes cell expansion in the grain hull and positively regulates the grain length and yield. OsSPL16 encoded by *GW8* on the other hand functions as a repressor of expression of *GW7* that encodes OsSPL13. Thus, the control of grain length is linked to expression of both *OsSPL13* and *OsSPL16*. The function of *OsSPL14* that encodes OsSPL14 is, however, not linked to the grain trait. Rather, it controls the panicle branching and the number of grains per panicle, the mechanistic details of which are yet to be known. The involvement of *OsSPL14* in determining panicle morphology is also indicated from the overexpression of miR164b-resistant *NAC2*, which leads to a better plant architecture with longer panicles and more grains compared with the non-transformed plant concomitant with upregulation of *IPA1* (Jiang et al., 2018). Overexpression of *NAC2* also leads to upregulation of *DEP1*, which also plays an important role in determining the panicle morphology in rice (Jiang et al., 2018).

Another plant-specific transcription factor is OsGRF4 (Growth-Regulating Factor 4), encoded by *LGS1* and *GS2*, which unlike OsSPL13 and OsSPL14 regulates both grain length and width (Hu et al., 2015; Chen et al., 2019). OsGRF4 interacts with the transcriptional co-activator OsGIF1 (GRF-interacting factor 1) (Li et al., 2016). As GIF1 has been reported to participate in the control of cell proliferation during leaf development (Kim and Kende, 2004), the interaction of OsGRF4 and GIF1 seems to be an important aspect of regulation of grain length and width in rice. The view is strengthened from the fact that the transgenic rice overexpressing *OsGIF1* produced larger and heavier grains than the wild type (Li et al., 2016).

### Post-Translational Regulation

Regulation of grain filling at the post-translational level has been reported first through the 14-3-3 protein interaction. The first line of evidence to this is the presence of 14-3-3 protein associated with the starch granules (Sehnke et al., 2000; Sehnke et al., 2001). Besides, SSIII family proteins have been found to carry the consensus motif for 14-3-3 binding. Furthermore, pull-down experiments considering His-tagged SS, SUS2, and AGPS show clear interactions with the GST-GF14f recombinant protein (Zhang Z. et al., 2019). A comparative study considering superior and inferior spikelets showing good and poor grain filling, respectively, shows that poor grain filling is associated with a greater expression of the 14-3-3 protein (You et al., 2017; Zhang Z. et al., 2019). The evidence for a negative role of the 14-3-3 protein in grain filling also stems from an RNAi study in which GF14f-RNAi plants showing reduction in expression of the 14-3-3 protein also showed a significant increase in grain weight and length (Zhang Z. et al., 2019). As opposed to the interaction of the 14-3-3 protein with the starch-biosynthesizing enzymes limiting grain filling, a major QTL, *GFR1* (*Grain filling rate 1*), has been mapped on the long arm of chromosome 10 that influences the grain filling by regulating the grain filling rate (Liu E et al., 2019). The candidate gene for *GFR1* was identified to encode the DUF461 domain protein of unknown functions that interacts with the Rubisco small subunit, leading to an increase in the grain filling rate, the mechanism of which is not yet clear (Liu M et al., 2019). However, it is known that Rubisco is an important enzyme determining the rate of carbon assimilation during photosynthesis, and thus, the interaction of the *GFR1* product with the enzyme might be increasing its carbon assimilation efficiency and the grain filling rate per se.

### Post-Transcriptional Regulation

#### Evidence From Differential Expression Studies

Post-transcriptional regulation of grain filling occurs through the action of miRNAs, which is reflected from several direct and circumstantial pieces of evidence. The circumstantial pieces of evidence of the regulatory role of miRNAs were initially reflected from their differential expressions in the caryopses during different stages of the grain development. Such differential expressions influenced the transcript abundance of *MYB*, *MADS*-box, *GRF*, *ARF*, and the *Brassinosteroid insensitive 1*-associated receptor kinase 1 precursor (*BAK1*), regulating various aspects of grain filling and development (Zhu et al., 2008; Xue et al., 2009; Peng et al., 2013; Yi et al., 2013). Later on, the differential expressions of miRNAs in the spikelets based on their spatial locations have also been studied in order to throw light on the possible influence of miRNAs in differential grain filling in the superior and inferior spikelets of compact and heavy panicles (Peng et al., 2011; Peng et al., 2014; Chandra et al., 2021; Panigrahi et al., 2021; Teng et al., 2021). These studies revealed that several miRNAs, including miR164/miR167, miR159, miR1861, and miR396h targeting auxin-responsive factor *ARF8*, *MYB*, beta-amylase, and auxin efflux carrier protein, respectively, were expressed higher in the poorly filled inferior spikelets compared with the well-filled superior spikelets, indicating negative regulatory role of these miRNAs in grain filling. It has also been shown that the miRNAs may regulate the grain filling positively as well. For example, miR819a in rice spikelets correlates positively with grain filling (Peng et al., 2011), and the loss of function of its target, an E3 ubiquitin ligase, accelerates the grain filling (Song et al., 2007), although the nature of the role of the protein in grain filling is yet to be known. Similarly, miR812f/miR812j also seems to have a positive regulatory role in grain filling, expressing more in the spikelets that are filled well and less in those filled poorly (Song et al., 2007). The target prediction revealed that the miRNA cleaves 1-aminocyclopropane-1-carboxylate oxidase (ACO), the enzyme that catalyzes the final step of ethylene biosynthesis. The finding fits well with the observation that the poorly filled spikelets produce more ethylene than the well-filled spikelets (Panda et al., 2015; Sekhar et al., 2015a; 2015b).

Chandra et al. (2021) reported six miRNAs, including osa-miR444e, osa-miR156c, osa-miR2118o, osa-miR12477, osa-miR1861, and osa-miR1436, that expressed significantly more in the poorly filled spikelets than that in the well-filled spikelets and emphasized that the poor filling of the grains could be linked to the cleavage of 1) the *MADS*-box transcription factor (the target of osa-miR444e) that plays an important role in seed development (Liu E et al., 2019), 2) *SPL19* (the target of osa-miR156c), an isoform of which (*SPL16*) controls grain size (Wang et al., 2012), 3) *pullalanase* (the target of osa-miR2118o), a starch-debranching enzyme responsible for the proper crystalline structure of the starch molecules, 4) *SS1* (the target of osa-miR12477 and osa-miR1436), which is involved in the extension of the ∝-1,4-polyglucan chain of the amylopectin, and 5) *ARF8* (the target of osa-miR164 and -miR167), which is possibly involved in rice grain filling by maintaining the cellular IAA level (Peng et al., 2014). The important regulatory role of miRNAs in grain filling is also reflected from differential miRNA expression in the Nipponbare rice variety in which moderate soil drying conditions (MD) improve grain filling in the otherwise poorly filled inferior spikelets (Teng et al., 2021). The improvement in grain filling in the inferior spikelets under MD accompanied significant upregulation of miR1861 and miR397, leading to a decrease in the transcript abundance of *OsSBDCP1* (encoding repressor of starch synthase IIIa) and *OsLAC* (Laccase), the negative regulators of *SSIIIa* expression and BR signaling, respectively, essential for grain filling (Teng et al., 2021). In contrast, the expression of miR1432 was downregulated in the inferior spikelets, resulting in upregulation of *OsACOT* (acyl-CoA thioesterase), consequently elevating the level of abscisic acid (ABA) and IAA, both playing a positive role in grain filling (Teng et al., 2021). The negative regulatory role of miR1432 in grain filling is also indicated from its higher expression in the poorly filled inferior spikelets compared with the well-filled superior spikelets of the compact panicle Mahalaxmi (Chandra et al., 2021).

#### Evidence From Developing Genetically Modified Plants

In somewhat direct evidence of miRNAs in grain filling, it has been found that the overexpression of osa-miR397 leads to an increase in seed size as a result of an increase in brassinosteroid signaling caused by downregulation of the *LAC* (*Laccase*) gene, the target of the miRNA (Zhang et al., 2013). The transgenic rice overexpressing *OsLAC* produced smaller grains than the wild type (Zhang et al., 2013), suggesting that *OsLAC* negatively influences grain size, in confirmation with the positive regulatory role of miR397 (Teng et al., 2021). However, the molecular mechanism as to how *LAC* regulates brassinosteroid signaling is yet to be known. In contrast to the *LAC* gene, enhanced expression of the Growth-Regulating Factor 4 (*OsGRF4*) leads to an increase in the grain size, both length and width, and the locus is kept under suppressed conditions by the action of osa-miR396c (Hu et al., 2015; Li et al., 2016). Another miRNA that negatively regulates grain size is osa-miR1432 that targets *OsACOT*. Transgenic experiments have also revealed that the downregulation of expression of osa-miR1432 increases the seed size significantly, while its overexpression decreases the seed size and the seed development (Zhao et al., 2019). The increase in the grain size was found to be because of an increase in the grain filling rate, probably because of an increase in the IAA and ABA levels as the miR1432-defective mutant and the OXmACOT plant whose miR1432 target site was mutated showed increased accumulation of both the hormones (Zhao et al., 2019). *OsACOT* encodes thioesterase protein, which is an enzyme that hydrolyses Acyl-CoA liberating free fatty acid. It was postulated that the downregulation of osa-miR1432 might be increasing the cellular free fatty acid, leading to an increase in the fluidity of the membrane favoring transport of auxins into the endosperm cells that might be promoting the grain filling (Liu et al., 2016; Zhao et al., 2019). In a similar but somewhat indirect relationship between miRNA expression and grain quality, it has been observed that the overexpression of miR1848 reduces the grain length compared with the wild type (Xia et al., 2015). Further study revealed that miR1848 targets *OsCYP51G3* encoding obtosifoliol 1,4-α-demethylase, which could be governing the transcript levels of *GS3* and *GS5* through brassinosteroid homeostasis (Xia et al., 2015), discussed above.

MiRNAs not only influence the grains trait but also the panicle morphology, including panicle branching and grain numbers. This is evident from the fact that the transgenic rice overexpressing osa-miR156b and -miR156h, which target several *OsSPL* genes, shows significant reduction in panicle size concomitant with delay in flowering (Xie et al., 2006). Subsequently, it was found that the *WFP* (*Wealthy farmer’s panicle*) locus in rice encoding *OsSPL14* carries a point mutation that abolishes the binding site to osa-miR156a, and the mutation is associated with increased panicle branching and grain yield in rice (Jiao et al., 2010; Miura et al., 2010). Besides, miR156, miR529, and miR535 also target *OsOSPL14* but at slightly shifted binding sites (Peng et al., 2019). The evidence of cleavage of *OsSPL14* by the two miRNAs comes from the plants overexpressing miR529a and miR535. These transgenic plants show smaller panicles with lesser grain numbers compared with the non-transformed plants (Wang L. et al., 2015; Sun et al., 2019). Furthermore, through the generation of miR529a overexpressing (miR529a-OE) and miR529a target mimicry (miR529a-MIMIC) transgenic plants, it has been seen that the miRNA negatively regulates panicle branching and grain numbers by altering the expression of five OsSPL genes, namely, *OsSPL2*, *OsSPL7*, *OsSPL14*, *OsSPL16*, *OsSPL17*, and *OsSPL18* (Yan et al., 2021). The presence of the miR164b target site on *NAC2* also keeps the expression of *NAC2* suppressed, which in turn keeps the expression of *OsSPL14* in control, finally leading to no ideal plant architecture (Jiang et al., 2018).

*OsGRF6* is another important gene that greatly influences panicle branches and the number of spikelets, as revealed from its overexpression (Gao et al., 2015). The loci are kept under suppressed conditions by the action of osa-miR396b, and the transgenic plants with reduced expression of miR396b show a significant increase in yield (Gao et al., 2015). OsGRF6 acts by directly binding with the promoter of *OsARF2* and *OsARF7* and the auxin-biosynthesis related genes like *OsYUCCA* (Gao et al., 2015). With regard to GRF, it has further been discovered that *OsGRF4*, *OsGRF6*, and *OsGRF8* are targeted by miR396e and miR396f, and the *mir396ef* mutants, generated by knockout of *MIR396ef* (*MIR396e* and *MIR396f*), showed an increase in grain size and increased panicle branching, suggesting the negative regulatory role of these miRNAs in grain development and panicle morphology (Zhang et al., 2020). Unlike that of *OsGRF*, *OsUCL8*, an uclacyanin (UCL) of the plastocyanin family, is a negative regulator of panicle branching and grain number, and in accordance, the overexpression miR408 that targets the gene results in increased panicle branching and grain numbers in the transgenic plant compared with the wild type (Zhang et al., 2017). It has been seen that *UCL8* affects copper homeostasis negatively, leading to a decrease in plastocyanin abundance required for photosynthesis (Zhang et al., 2017). However, any relationship between expression of the gene and panicle morphology is yet to be delineated.

Sometimes, a single miRNA may be involved in regulating the expression of two genes combined in determining a phenotype as that of *SNB* and *OsIDS1* determining the inflorescence structure and panicle morphology (Lee and An, 2012). Both the genes are targeted by miR172, and overexpression of the miRNA results in severe defects in the phenotype concomitant with significant reduced expression of *SNB* and *OsIDS1* (Lee and An, 2012).

### Hormonal Control of Grain Filling

**FIGURE 7:**
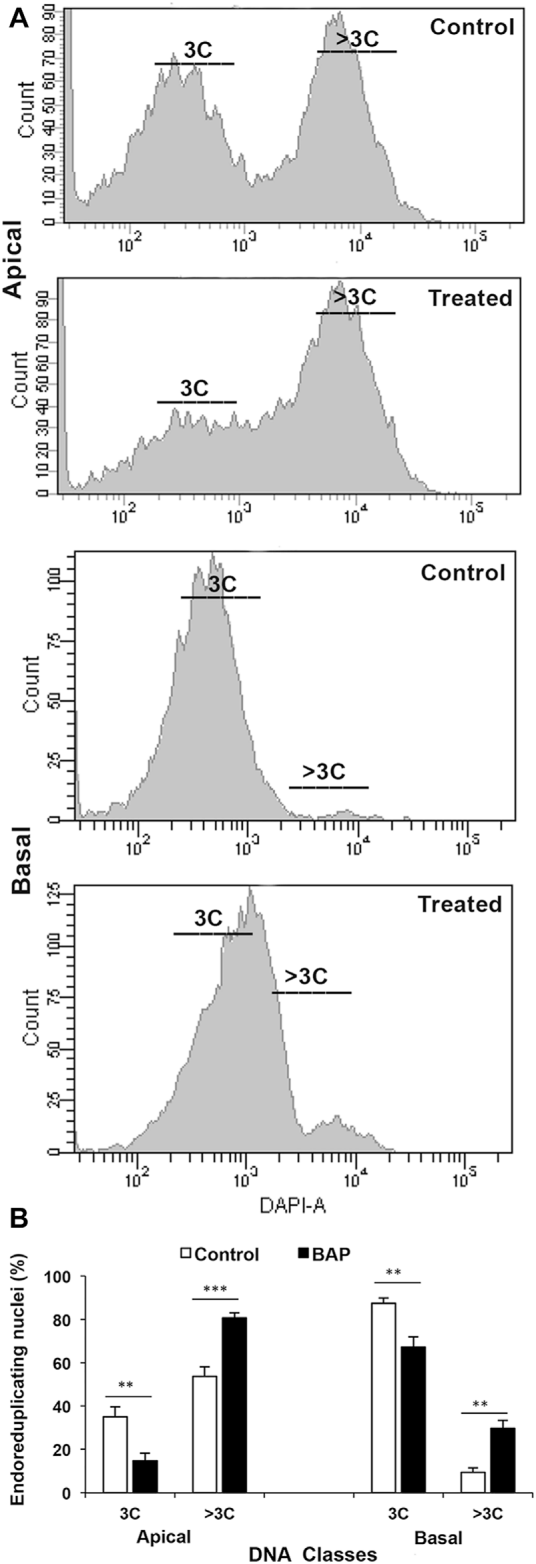
Ploidy status (DNA class) of the endosperm nuclei of the apical and basal spikelets of a compact-panicle rice cultivar, OR-1918, sampled on the 9 days after fertilization from the control plant and that sprayed with 6-benzylaminopurine (BAP) during the heading. **(A)** The endosperm nuclei ploidy status is much lesser in the caryopses of the basal spikelet compared with that in the apical spikelets. **(B)** Upon the BAP application, the ploidy status of the endosperm nuclei in the basal spikelets increased significantly. Reproduced with the permission of the author (Panda et al., 2018).

Plant hormones play important roles in all aspects of the plant development, including grain filling, some details for which are available for auxins, gibberellins, cytokinins, ABA, brassinosteroids, and ethylene. A large transient increase in the concentrations of cytokinins (CKs), gibberellins (GAs), IAA, and ABA observed in the endosperm liquid during grain development is indicative of their important role in grain filling (Yang et al., 2000; Eeuwens et al., 1975; Lur and Setter, 1993; Kato et al., 1993). Several other studies also elaborate important roles of the plant hormones in grain filling: 1) the levels of CKs and IAA reach to their maximum values just before the grain filling rate becomes the maximum and the endosperm cell division rate is at its peak (Yang et al., 2001), 2) the level of ABA in the endosperm cells reaches to the maximum level at the mid- and late-mid-grain filling stage and positively correlates with 14C partitioning, suggesting that the hormone mobilizes carbon assimilates into the grain during the grain filling (Yang et al., 2001), 3) application of CKs at the early stage of grain development increases the endosperm cell numbers and cell area, much required for efficient grain filling (Yang et al., 2003; Panda et al., 2018), 4) the rice spikelets that show good grain filling contain a higher level of CKs (Figure 3), IAA, and ABA compared to that showing poor grain filling (Zhang et al., 2009; Panda et al., 2018), and 5) CK increases the ploidy level of the endosperm cells and improves grain filling in the otherwise poorly filled basal spikelets in rice (Panda et al., 2018) (Figure 7).

The maintenance of the level of at least two of the plant hormones, namely, ABA and cytokinin, in the caryopses by decreasing their degradation rather than by promoting their synthesis has also been shown to play important roles in grain and panicle development. The best known among these is the prevention of breakdown of cytokinin through the downregulation of expression of cytokinin oxidase (*OsCKX2*) mediated by the loss of function mutation of *Gn1a* (Ashikari et al., 2005) and *LP* (Li M. et al., 2011). It has also been observed that under moderate soil drying conditions, the individual grain weight of the inferior spikelets is increased significantly compared with the well-watered control concomitant with nearly 50% increase in the level of ABA in the caryopses, and the increase in the level of ABA accompanied nearly a 5-fold decrease in the expression of *ABA8OX2*, an ABA oxidase (Wang et al., 2019).

Several recent studies have given more emphasis to the role of the plant hormones in carbon resource remobilization from the straw to the grains rather on the development of the grain per se in enhancing the rice yield. It has been stressed upon that remobilization of the carbon reserves from the straw to the grains is very important in grain filling in rice, and a higher ABA level in the straw favors this remobilization (Wang et al., 2020a, 2020b; Wang and Zhang, 2020). Although the mechanistic details of role of ABA in the carbon resource remobilization are not known, the level of ABA increased in the straw as much as by 10 times under moderate soil drying conditions that favored grain filling compared with that under the well-watered control (Wang et al., 2020b). The increase in the level of ABA accompanied a significant decrease in the expression of *ABA8OX1* and *ABA8OX2*, both involved in degradation of the hormone (Wang et al., 2020a; 2020b). Furthermore, the level of ABA in the straw has also been related to the grain filling in the inferior spikelets as the conventional rice in which the grain filling is proper in the inferior spikelets contains a higher level of ABA in the straw compared with the super rice that shows poor filling of grains in the inferior spikelets (Wang et al., 2017).

Similar to ABA, brassinosteroid (BR), which is comparatively a recent addition in the plant hormones influencing panicle morphology, also appears to significantly influence grain filling through remobilization of the carbon resources from the straw to the grains (Wang et al., 2020b). This is reflected from a significant increase in the expression of the gene of brassinosteroid receptor kinase-interacting protein 135 as well as of the protein itself in the straw during the grain filling stage under the moderate soil drying conditions that improve grain filling in the inferior spikelets (Wang et al., 2017; Wang et al., 2020b). However, the functional significance of expression of this protein in regulation of the carbon resource remobilization is not yet clear.

**FIGURE 8:**
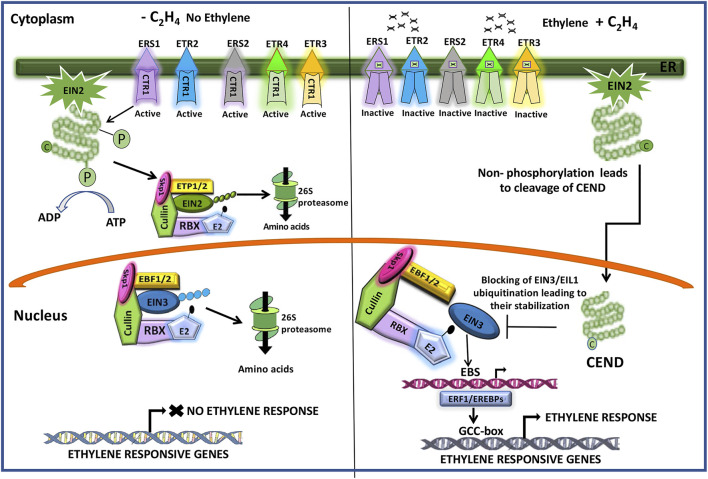
Cartoon showing the molecular mechanism of ethylene action. The histidine kinase domain of CTR1 (constitutive triplet response1) of the ethylene receptors (ERS1, ERS2, ETR2, ETR3, ETR4) keeps on phosphorylating the CEND (C-terminal end) of the membrane bond protein EIN2 (ethylene insensitive2). This follows its recognition by the F-box proteins ETP1/2 (EIN2 targeting protein1/2) for ubiquitination by the SCF (Skp1-Culin-F-box) E3 ligase complex that additionally contains RBX, a RING box protein, and the E2 ubiquitin-conjugating enzyme and degradation of the ubiquitinated protein by the 26S proteosome system. Upon binding of ethylene to the receptors, the CTR1 kinase activity is inhibited, and the CEND gets detached and moves to the nucleus where it blocks the ubiquitination of EIN3 (ethylene insensitive3)/EIL1 (ethylene insensitive3 like1), preventing its 26S proteosomal degradation. Accumulation of EIN3 promotes it to bind to the EBS (Ein3/EIL1-binding site) in the promoter of the ethylene responsive factor1 (ERF1) and other ethylene-responsive element binding proteins (EREBPs) to drive their expression. ERF1/EREBPs in turns bind to the GCC-box in the promoter of the ethylene-responsive genes, leading to ethylene response. In the absence of EIN2 in the nucleus, EIN3 is recognized by the F-Box proteins EBF1/2 (EIN3-binding F-Box Protein1/2) for ubiquitination through the SCF (Skp1-Culin-F-box) E3 ligase complex, followed by its degradation by the 26S proteosome. Adapted from Ji and Guo (2013).

Unlike the other plant hormones, ethylene is gaseous in nature and plays an inhibitory role in grain filling in rice (Sekhar et al., 2015a, 2015b; Panda et al., 2015; Das et al., 2016; Sahu et al., 2021). The hormone rapidly produces its effect as it diffuses in and out of the cells freely. The ethylene signal is perceived by plants through the endoplasmic membrane-bound receptors, including ERS1, ERS2, ETR2, ETR3, and ETR4, that contain the histidine kinase domain, CTR1 (Constitutive Triplet Response1), at the cytoplasmic side. The presence of ethylene inhibits the kinase activity of CTR1, leading to detachment of the C-terminal end (CEND) of the membrane-bound protein EIN2 that is phosphorylated otherwise. The EIN2 C-terminus (CEND) moves to the nucleus where it regulates the expression of *EIN3* and *EIL1* (*EIN3-like*). EIN3 activates the transcription of an ethylene-responsive element binding protein (*EREBP*) transcription factor, *ERF1* (ethylene responsive factor1), and other *EREBPs*, the products of which in turn interact with the GCC-box present in the promoter of other ethylene-responsive genes and regulate their expression (Figure 8).

**FIGURE 9:**
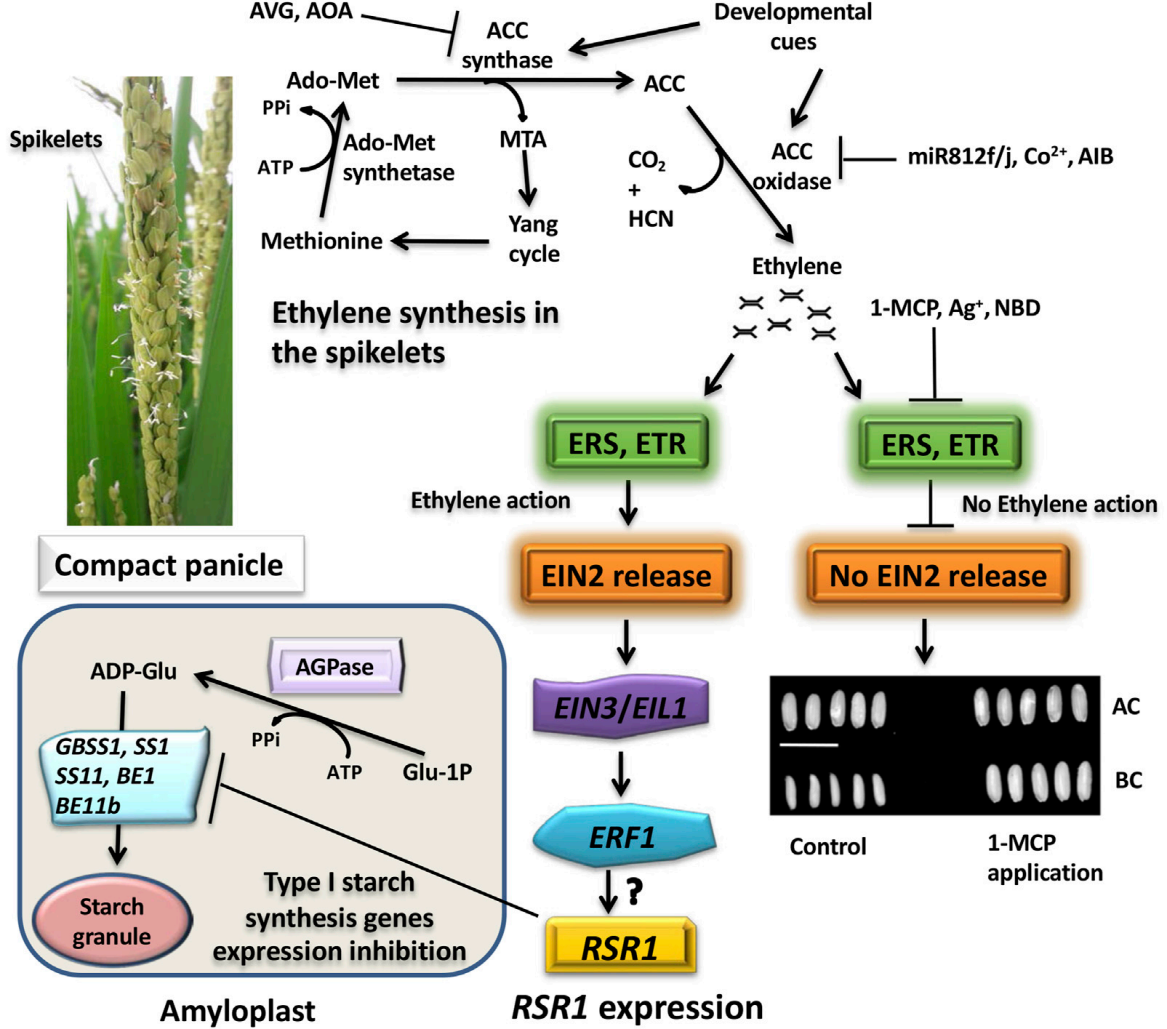
Pictorial presentation of ethylene biosynthesis and ethylene action. Ethylene is synthesized from methionine by the action of ACC synthase that forms ACC (1-aminocyclopropane-1-carbooxylic acid), which is catalyzed by ACC oxidase (ACO) to yield ethylene and HCN as the byproduct. Methionine is regenerated via the Yang cycle. Ethylene action leads to the synthesis of Rice Starch Regulator1 (RSR1), which reportedly inhibits the expression of the type I starch-biosynthesizing enzymes, leading to inhibition of starch biosynthesis and poor grain filling. Inhibition of ethylene synthesis by AVG (2-aminoethoxyvinyl glycine), AOA (2-aminoooxyacetic acid), AIB (aminoisobutyric acid), Co^2+^, and the miRNAs miR812f/j and blocking of the ethylene action by the use of ethylene receptor blockers like 1-MCP (1-methylcyclopropene), Ag^+^, and NBD (2,5-norbornadiene) lead to no ethylene action resulting in proper filling of the grain. MTA, methylthioadenine; Ado-Met, adinosyl-methionine; ERS, ETR, ethylene receptors; AC, apical caryopses; BC, basal caryopses.

Although hypothesized, the inhibitory role of ethylene in grain filling is not understood well and is only reflected from the circumstantial pieces of evidence, such as the following: 1) the inferior spikelets of the compact panicle of rice showing poor grain filling produce more ethylene than the superior spikelets producing well-filled grains (Panda et al., 2015; Sekhar et al., 2015a; 2015b; Das et al., 2016); 2) application of ethylene synthesis inhibitors like cobalt nitrate on the panicle at the initiation of heading significantly increases the grain filling in the inferior spikelets of the compact panicle (Mohapatra et al., 2000); 3) application of CEPA (2-chloroethylphosphonic acid), an ethylene-releasing compound, on the panicle at heading reduces the grain filling in the spikelets (Naik and Mohapatra, 2000); 4) application of 1-MCP, a blocker of ethylene receptors, on the panicle during heading leads to a significant increase in grain filling in the inferior spikelets producing a significantly higher amount of ethylene compared with the superior spikelets (Panda et al., 2016a; Zhang et al., 2015); 5) the spikelets of the compact-panicle cultivars, particularly the inferior ones showing poor grain filling, show higher expression of the ethylene receptors than that of the open-panicle cultivars, indicating that the response to ethylene is greater in the former than in the latter and hence the poor grain filling (Sekhar et al., 2015a); and 6) overexpression of *ETR2* results in reduced seed setting and a decrease in the thousand grain weight, whereas knockdown of the receptor by RNAi results in an increase in the thousand grain weight (Wuriyanghan et al., 2009). In addition, it has been seen that the compact panicle showing poor grain filling shows a greater expression of the downstream ethylene signaling components, such as *ERF2*, *ERF3*, and *EREBP5*, compared with the lax panicle showing good grain filling (Sekhar et al., 2015a). Moreover, in the compact panicle, the expression of *RSR1* (rice starch regulator1), an APETALA2/EREBP family transcription factor that shows a negative relationship with the expression of *GBSS1*, is higher in the inferior spikelets showing poor grain filling compared with the superior spikelets showing good grain filling (Sekhar et al., 2015a; Panda et al., 2016b). Overall, the inhibitory role of ethylene in grain filling in rice is well documented (Figure 9). In addition, the inhibitory role of the ethylene in grain filling can be perceived from the fact that CN^−^ is formed as a byproduct during ethylene biosynthesis (Machingura et al., 2016), and it is well known that cyanide is a potent inhibitor of mitochondrial electron transport (Solomonson, 1981). The inhibitory role of ethylene on the mitochondrial electron transport is reflected from a comparative analysis of the JC-1 fluorescence signal from caryopsis of the inferior and superior spikelets of a compact panicle, with the former showing lesser JC-1 staining than the latter (Sekhar et al., 2015b). A lower JC-1 staining is suggestive of inhibition of the mitochondrial electron transport, and thus a poor generation of ATP, in the inferior spikelets compared with the superior ones showing intense JC-1 staining (Sekhar et al., 2015b). Since starch synthesis is an ATP-consuming process, its synthesis is likely to be inhibited in the inferior spikelets in which the mitochondrial electron transport is inhibited (Sekhar et al., 2015b).

## Conclusion and Perspectives

It is well recognized that rice is a staple crop and its production must increase with the increase in the population of the world. The undergoing research studies the world over focusing on increasing the rice yield have also raised concern, which the scientific community has on near stagnation of the rice production during the past decade (https://www.statista.com/statistics/271972/world-husked-rice-production-volume-since-2008/). So far, the green revolution in rice production the world has experienced is only through breeding and advanced agricultural practices. The two modern pillars of applied research, molecular biology and biotechnology, have so far contributed little in increasing the rice yield, despite our current in-depth understanding on the biochemistry, molecular biology, and genetics of the yield characteristics in rice. An important phenomenon related to grain filling that has been discovered is the positive correlation between grain filling and the inter-spikelet distance, that is, the grain filling is poor, particularly in the basal spikelets, when the panicle is compact bearing numerous spikelets (Sahu et al., 2021). Although the spikelet thinning treatment of heavy panicles confirms that all the spikelets, including the poorly filled inferior ones, are genetically competent to develop into well-filled grains (Kato, 2004; You et al., 2016), it does not provide any information on if the poor grain filling was related to a low inter-spikelet distance. The discovery that the panicle branching is greatly regulated by the genes like *Gn1a*, *APO1*, *LOG*, *DEP1*, *RNC1*, *RNC2*, and so forth, nevertheless, does indicate genetic control on the inter-spikelet distance. Hence, it is perceivable that the inter-spikelet distance is genetically controlled. If so, the identification of such gene(s) would provide the researchers a chance to manipulate the inter-spikelet distance through biotechnological interventions, and hence, it might be possible to convert a compact panicle into an open architecture without altering the number of spikelets borne on them. Considering the fact that an inter-spikelet distance greater than 0.5 cm favors grain filling, converting a compact panicle into an open architecture should certainly lead to improvement in grain filling. In such a case, it is highly possible that the production of rice could be increased by as much as 30% as in a compact panicle, more than 30% of the spikelets remain unfilled. Thus, it would be possible to achieve the production target set for the year 2050. Working on the hypothesis, breeding is in progress in our lab for identification of the QTLs for panicle compactness. It is also possible to increase the production of the crop by taking a targeted molecular biology approach to increase the grain size and weight as currently, our knowledge on the genes regulating these traits is also sufficiently enough for such an attempt. However, although such an approach may lead to an increase in rice production, it would be of limited implication as the demand of rice is greatly dependent on its quality, which might be compromised. On the other hand, if rice production is increased by increasing grain filling in the heavy and compact-panicle cultivars producing grains of desired quality, it would be possible to achieve both the quality and the production target. In this regard, the discovery of a positive relationship between the levels of ABA and BRs in the straw and the carbon resource remobilization from the straw to the grains leading to improvement of grain filling in the inferior spikelets (Wang et al., 2020a; 2020b) is of immense significance. On this basis, genetic manipulation leading to an increase in the synthesis of the hormones in the straw, particularly during the grain filling stage, could increase the rice yield significantly. To achieve this, the enzymes acting as the rate-limiting step in the synthesis of these hormones may be identified and their genes may be overexpressed using the promoters that become active in the straw during the grain filling period.

The modern biotechnological intervention techniques can also be utilized in many other ways for increasing the rice production. First, as the expressions of the starch-biosynthesizing enzymes have been noted to be significantly less in the poorly filled inferior spikelets (Panda et al., 2015), their overexpression, particularly of *SUS*, using a seed-specific promoter could be of much help in increasing the grain filling of these spikelets and the rice production per se. Second, increasing scavenging of the CN^−^ formed during ethylene biosynthesis by seed-specific overexpression of *β-CAS*, the scavenger of CN^−^, may improve the grain filling significantly as CN^−^ is a potent inhibitor of enzyme activity. Work on this line is also in progress in our lab. Third, and most importantly, since ethylene biosynthesis is supposedly the root cause of inhibition of grain filling, genetic manipulation may be considered in reducing the ethylene biosynthesis itself for improvement in grain filling. One way of approaching a solution to the problem would be the spikelet/seed-specific RNAi-mediated silencing of ACO catalyzing the final and crucial step of ethylene biosynthesis. Besides, the synthesis of ethylene can also be reduced by spikelet/seed-specific overexpression of miR812f,j that targets the product of *ACO*. The spikelet/seed-specific inhibition of ethylene biosynthesis would not only lead to a decrease in accumulation of the toxic CN^−^ but also lessen the level of ethylene signaling components, such as RSR1, believed to inhibit the expression of the type I starch-biosynthesizing enzyme. Keeping in view the stagnation of rice production since the past decade and inability of the breeders to improve the rice yield further substantially, biotechnological intervention is probably the only way left out in increasing the rice yield further to achieve the production target by the year 2050.
