# Supplementary material for: Effect of Panicle Morphology on Grain Filling and Rice Yield: Genetic Control and Molecular Regulation
Source: Front Genet. 2022 May 10;13:876198. doi: 10.3389/fgene.2022.876198 (PMC9127237; doi:10.3389/fgene.2022.876198)
Supplement: Supplementary file 1 [file Table1.DOCX]

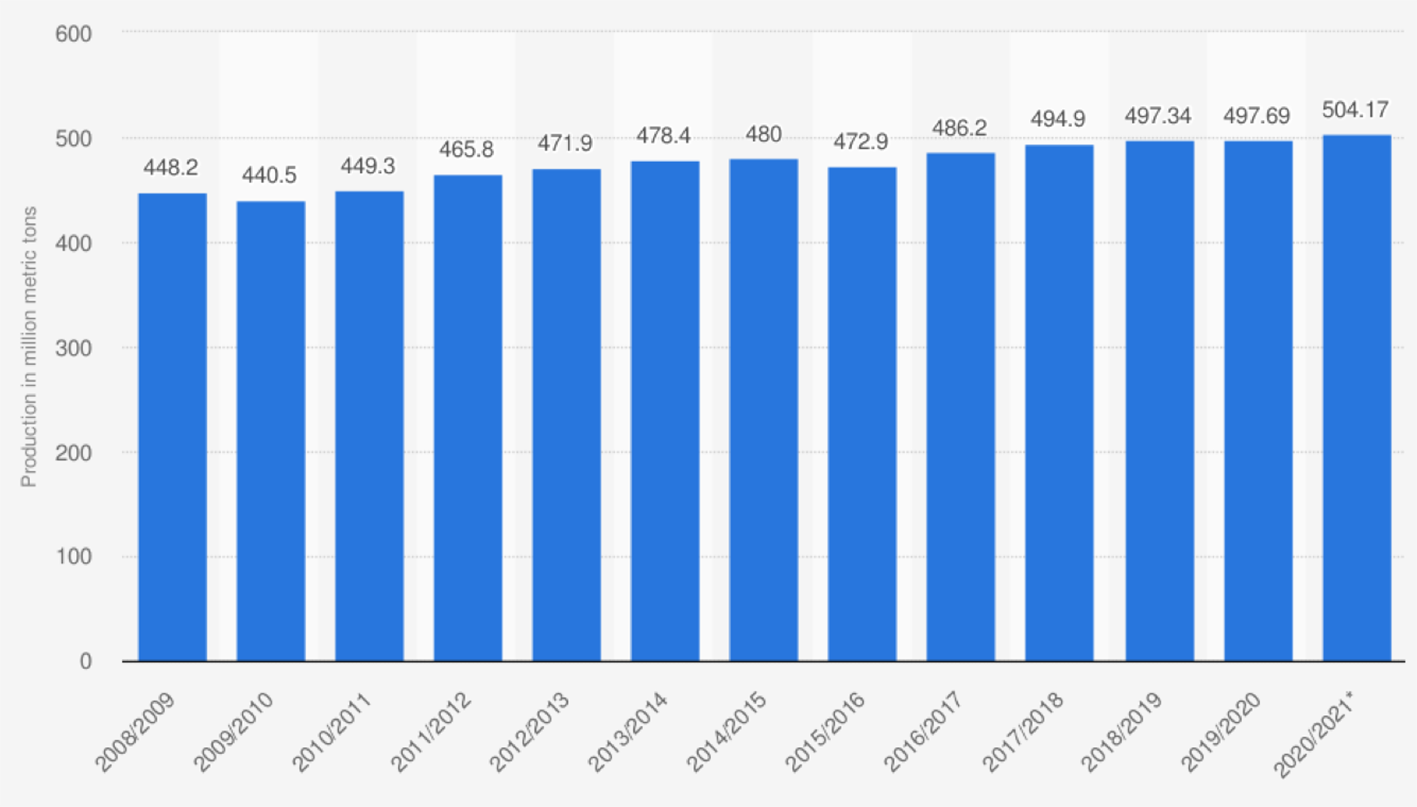


**Fig. S1**. Current scenario of world rice production. The rice production is getting increasingly flattened, threatening increase in gap between demand and supply in future. Adapted from [9].
