# Supplementary material for: Effect of Panicle Morphology on Grain Filling and Rice Yield: Genetic Control and Molecular Regulation
Source: Front Genet. 2022 May 10;13:876198. doi: 10.3389/fgene.2022.876198 (PMC9127237; doi:10.3389/fgene.2022.876198)
Supplement: Supplementary file 2 [file Table2.DOCX]

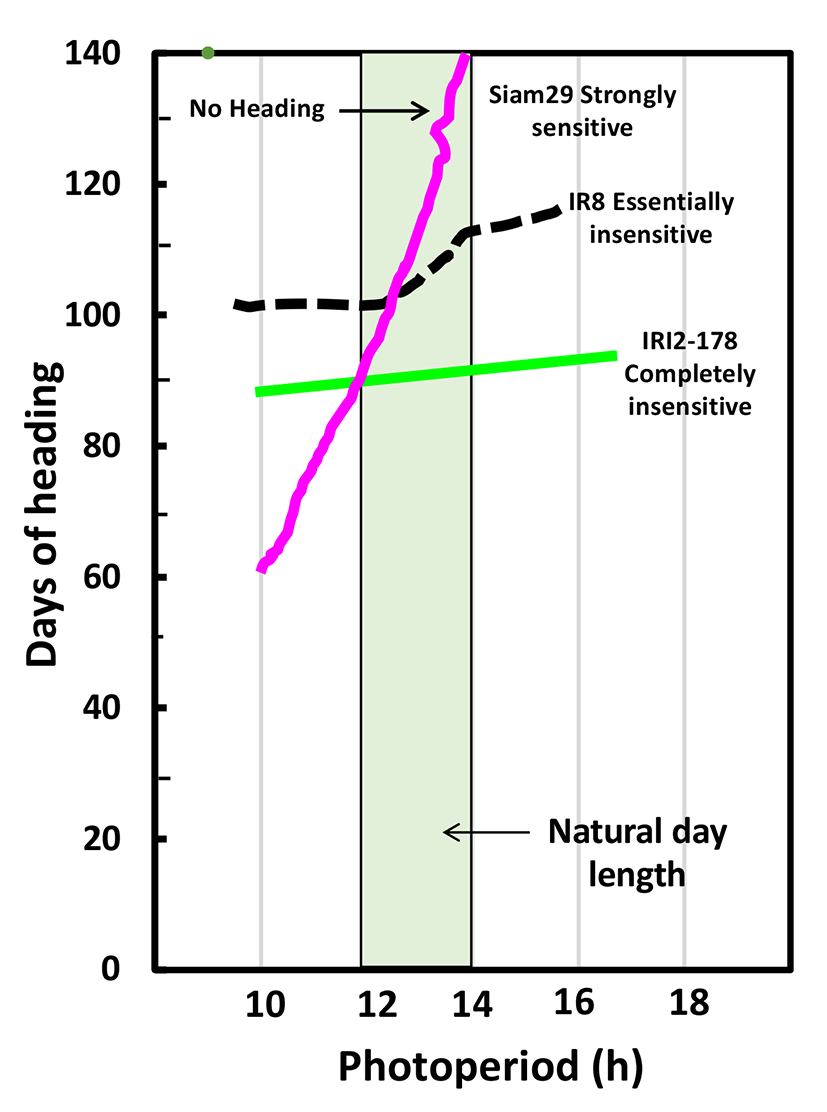


**Figure S2. Pictorial representation of sensitiveness of three rice varieties to photoperiod.** IR8 varieties that brought green revolution are essentially photo-period insensitive. Adapted from Vergara and Chang, 1985.
